# Betaine delays age‐related muscle loss by mitigating Mss51‐induced impairment in mitochondrial respiration via Yin Yang1

**DOI:** 10.1002/jcsm.13558

**Published:** 2024-08-26

**Authors:** Si Chen, Tongtong He, Jiedong Chen, Dongsheng Wen, Chen Wang, Wenge Huang, Zhijun Yang, Mengtao Yang, Mengchu Li, Siyu Huang, Zihui Huang, Huilian Zhu

**Affiliations:** ^1^ Department of Nutrition, School of Public Health Sun Yat‐sen University Guangzhou China; ^2^ School of Public Health, Guangdong Provincial Key Laboratory of Food, Nutrition and Health Sun Yat‐sen University Guangzhou China; ^3^ Department of Hepatobiliary Oncology, State Key Laboratory of Oncology in South China, Sun Yat‐sen University Cancer Center Sun Yat‐sen University Cancer Center Guangzhou China; ^4^ Center of Experimental Animals Sun Yat‐sen University Guangzhou China

**Keywords:** Betaine, Mitochondrial dysfunction, Mss51, Muscle loss, Transcription factor, Yin yang1 (Yy1)

## Abstract

**Background:**

Mitochondrial dysfunction is one of the hallmarks of aging and a leading contributor to sarcopenia. Nutrients are essential for improving mitochondrial function and skeletal muscle health during the aging process. Betaine is a nutrient with potential muscle‐preserving properties. However, whether and how betaine could regulate the mitochondria function in aging muscle are poorly understood. We aimed to explore the molecular target and underlying mechanism of betaine in attenuating the age‐related mitochondrial dysfunction in skeletal muscle.

**Methods:**

Young mice (YOU, 2 months), old mice (OLD, 15 months), and old mice with betaine treatment (BET, 15 months) were fed for 12 weeks. The effects of betaine on muscle mass, strength, function, and subcellular structure of muscle fibres were assessed. RNA sequencing (RNA‐seq) was conducted to identify the molecular target of betaine. The impacts of betaine on mitochondrial‐related molecules, superoxide accumulation, and oxidative respiration were examined using western blotting (WB), immunofluorescence (IF) and seahorse assay. The underlying mechanism of betaine regulation on the molecular target to maintain mitochondrial function was investigated by luciferase reporter assay, chromatin immunoprecipitation and electrophoretic mobility shift assay. Adenoassociated virus transfection, succinate dehydrogenase staining (SDH), and energy expenditure assessment were performed on 20‐month‐old mice for validating the mechanism *in vivo*.

**Results:**

Betaine intervention demonstrated anti‐aging effects on the muscle mass (*P* = 0.017), strength (*P* = 0.010), and running distance (*P* = 0.013). Mitochondrial‐related markers (ATP5a, Sdha, and Uqcrc2) were 1.1‐ to 1.5‐fold higher in BET than OLD (all *P* ≤ 0.036) with less wasted mitochondrial vacuoles accumulating in sarcomere. Bioinformatic analysis from RNA‐seq displayed pathways related to mitochondrial respiration activity was higher enriched in BET group (NES = −0.87, FDR = 0.10). The quantitative real time PCR (qRT‐PCR) revealed betaine significantly reduced the expression of a novel mitochondrial regulator, Mss51 (−24.9%, *P* = 0.002). In C2C12 cells, betaine restored the Mss51‐mediated suppression in mitochondrial respiration proteins (all *P* ≤ 0.041), attenuated oxygen consumption impairment, and superoxide accumulation (by 20.7%, *P* = 0.001). Mechanically, betaine attenuated aging‐induced repression in *Yy1* mRNA expression (BET vs. OLD: 2.06 vs. 1.02, *P* = 0.009). Yy1 transcriptionally suppressed *Mss51* mRNA expression both *in vitro* and *in vivo*. This contributed to the preservation of mitochondrial respiration, improvement for energy expenditure (*P* = 0.008), and delay of muscle loss during aging process.

**Conclusions:**

Altogether, betaine transcriptionally represses *Mss51* via Yy1, improving age‐related mitochondrial respiration in skeletal muscle. These findings suggest betaine holds promise as a dietary supplement to delay skeletal muscle degeneration and improve age‐related mitochondrial diseases.

## Introduction

Sarcopenia is characterized by the progressive loss of skeletal muscle mass, strength, and function during the aging process,^1^ resulting in reduced mobility, fragility, and disability in the aging populations. The high prevalence of sarcopenia in older adults has emerged as a significant public health concern and strategies to combat muscle loss are warranted as early as possible to promote healthy aging in society.^2^ Aging leads to a reduction in both the overall number of mitochondria and the expression of proteins related to mitochondrial respiration, which is one of the major contributor to muscle loss.^3^ During aging, there is an approximate 15% decline in the expression of muscle mitochondrial respiration‐related proteins (e.g., NDUFA9 and SDHA).^4^ Meanwhile, age‐related blunt in the muscle mitochondrial oxidative phosphorylation (OXPHOS) leads to the accumulation of aberrant mitochondrial vacuoles and reactive oxygen species (ROS), subsequently impairing the sarcomere structure and exacerbating muscle loss.^5^ Moreover, mitochondrial respiration inhibition results in diminished energy metabolism, as evident by reduced VO_2max_ and substantial impairment in lower limb strength, ultimately impeding mobility in aging individuals.^6^ Hence, approaches mitigating age‐related mitochondrial dysfunction hold promise in preventing against age‐related muscle loss.

Betaine is a dietary methyl donor that can be sourced from beetroot, wheat and shrimp shells.^7^ Epidemiological studies displayed the positive associations between the dietary betaine intake and the preservation of the lean mass in the middle‐age and older adults.^8^ Our previous animal study demonstrated that betaine promotes protein synthesis and preserves skeletal muscle mass and strength.^9^ These results suggested dietary betaine supplementation is a promising approach for delaying the age‐related muscle loss. Additionally, several *in vitro* or *in vivo* studies have indicated betaine has the potential to regulate mitochondrial function. For examples, betaine‐treated H2.35 cells exhibited upregulated mitochondrial respiration and cytochrome c oxidase activity.^10^ In an acute liver injury model, betaine demonstrated hepatic protective effects by enhancing mitochondrial fusion and cellular survival.^11^ Additionally, betaine could increase mitochondrial content and facilitate the hepatic fatty acid beta oxidation to regulate the lipid metabolism.^12^ Nevertheless, the effects and underlying mechanisms of betaine on improving mitochondrial function in skeletal muscle during the aging process remain unclear.

A skeletal muscle‐specific mitochondrial regulator, Mss51, can comprehensively affect assembly of mitochondrial electronic transport chain (ETC),^13^ regulate ROS,^14^ and control mitochondrial biogenesis.^15^ These findings indicate that Mss51 may be a promising target for regulating mitochondrial function in aging muscle. Recent studies suggested that Mss51 could be transcriptionally controlled.^16^ Furthermore, a molecular docking and simulation study suggests that Mss51 could be a target for type 2 diabetes by interacting with zinc‐related natural compounds.^17^ Betaine showed promise in the regulation of gene transcription.^18^ Additionally, its methyltransferase (BHMT) is a zinc (Zn)‐dependent methyltransferase.^19^ As a natural dietary component, few evidence showed the effects of betaine on Mss51.

Given these observations, we explored effects and mechanism of betaine on ameliorating age‐related mitochondrial dysfunction. Our findings will suggest that betaine holds potential as a novel anti‐aging treatment, offering a promising alternative to genetic reprogramming technologies while avoiding their associated complexities.

## Methods

### Mice and intervention

Male C57BL/6J mice aged 2 months and 15 months were obtained from Guangdong Animal centre and housed on a 12 h light/dark cycle. The mice were divided into three groups: young group (YOU, 2 month), old group (OLD, 15 months) and old group treated with betaine (BET, 15 months). All mice were fed *ad libitum* with a standard chow diet for 12 weeks. The mice in the YOU and OLD were given distilled water, while the mice in the BET group were given distilled water with 2% (*w*/*v*) betaine. The animal experiment has been approved by the ethics committee of school of public health, Sun Yat‐sen University (Permit No. 2017‐007).

### Body composition

Body composition analysis was conducted using a Small Animal MRI System (Niumag, NM21‐060H‐I, China) following the manufacturer's instructions.

### Grip strength

Muscle strength was evaluated using a grip test with a grip strength meter (ZS‐ZL, China). The mice were placed on a metal bar with all four limbs until they calmed down. The peak grip strength was recorded using the MiniTAR system.

### Running test

Mice underwent a running test on a treadmill (ZS‐PT‐III, China) for 15 min, starting at a speed of 10 m/min and increasing to 25 m/min over 2 min. The intensity of the electronic current was set as 0.5 mA. The results of running test were assessed based on the distance covered, time taken, and speed achieved by the mice.

### Muscle histological analyses

After assessing muscle mass and strength, the mice were sacrificed, and their gastrocnemius muscles were isolated for H&E staining and transmission electron microscopy (TEM). Detailed procedures are described in Data S1 and S2.

### Gene expression analysis

Total RNA was extracted from approximately 30 mg of gastrocnemius muscle by homogenizing (TissueLyser II, QIAGEN) in TRIzol (Invitrogen, USA) and reverse transcribed using the PrimeScript™ RT reagent kit (TAKARA, Japan). The gene expression analysis was performed using a qRT‐PCR system with SYBR green fluorescence (TAKARA, Japan), with data normalized to *18s* expression. Primer sequences are provided in Table S1.

### Western blotting

The total protein lysates were extracted from gastrocnemius muscle or cells in RIPA with 2× protease complete cocktail (Beyotime, China). The total protein was quantitated by the bicinchoninic acid assay (BCA, Soyotime) method. The protein sample was mixed with loading buffer (1:4, v:v) and denatured at a temperature of 100°C. Equal amount of protein sample were resolved on a SDS‐PAGE gel system (8%, 10%, or 12%, Bio‐Rad) and transferred to PVDF membrane (MilliporeSigma). The primary antibodies and secondary antibodies were incubated sequentially. The proteins were finally visualized by TANON imaging system with the Signal Fire ECL reagent (Invitrogen). The semi‐quantifications were then performed by the ImageJ software (version 1.52a, NIH, USA) after normalizing the total protein amount, Gapdh or Vdac1 expression. Primary antibodies were Sdha (Abcam, ab137040), Vdac1 (Abcam, A19707), Ndufb8 (Abcam, ab192878), Atp5a (Abcam, ab176569), Uqcrc2 (Abcam, ab203832), MtCO1 (Abcam, ab203912), and Gapdh (CST, #2118).

### RNA sequencing

Approximately 100 mg gastrocnemius muscle from mice in the three groups were applied for RNA sequencing underwent RNA sequencing performed by GENEWIZ™ (Azenta Life Sciences, Suzhou, China) following standard procedures for library construction and annotation.

### Cell lines

C2C12 cells (National Collection of Authenticated Cell Cultures, China) were cultured at 37°C with 5% CO_2_ in DMEM supplemented with 10% fetal bovine serum (FBS) and 1% penicillin–streptomycin. When the confluency of cells intensity reached to 85%, the medium was changed into DMEM supplemented with 3% horse serum for 3 days for differentiation.

### Construction and transfection of lentiviral vectors

For stable transduction of Mss51 in C2C12 cell, the lentivirus‐induced overexpression plasmid vector (pSLenti‐EF1‐EGFP‐P2A‐Puro‐CMV‐3xFLAG‐WPRE) was constructed by the OBIO (Inc, Shanghai, China). The detail was described in Data S1.

### Mito superoxidase production

Mitochondrial superoxide accumulation was assessed using the MitoSOX™ Red reagent (Invitrogen) as the manufacturer instruction in C2C12 cells.

### Seahorse oxygen consumption rate analysis

Cellular respiration was evaluated using the Seahorse XFe96 Analyser (Agilent, USA) and the Seahorse Mitochondrial Stress Test kit as described in Data S1.

### Assessment of interaction between transcription factor and transcription‐start‐site

#### Luciferase reporter assay

The 2‐kb region upstream of the Mss51 promoter region was predicted using UCSC database (http://genome.ucsc.edu) and JASPAR database. The detail for the luciferase assay was describe in Data S1.

#### Chromatin immunoprecipitation

Chromatin immunoprecipitation (ChIP) was performed following the protocol of Pierce Magnetic ChIP Kit (Thermo Scientific™, USA). The procedure is detailed in Data S1.

#### Electrophoretic mobility shift assay (EMSA)

The electrophoretic mobility shift assay (EMSA) was conducted according to the LightShift™ EMSA kit manual (Thermo Scientific™, USA), with details in Data S1.

### Skeletal muscle specific transfection

Mice aged 16 months were purchased from Guangdong Yaokang (SYXK 2017‐0080) and divided into WT, 2%BET, Yy1^SM‐OE^, and Yy1^SM‐KD^ groups. All mice were raised to 20 months. At the age of 19 months, the adenoassociated virus (AAV) of different expression of Yy1 vectors was transfected into the mice TA muscle (Figure 6A). The animal experiment has been approved for the ethics committee of school of public health, Sun Yat‐sen University (Permit No. 2022‐018).

### Thermogenesis experiments

At the age of 20 months, all mice underwent thermogenesis experiments as per the previous literature.^20^ The data were then analysed by a web‐based analysis tool, *CalR* (https://CalRapp.org/).

### Statistical analysis

Statistical analyses were performed with R software (version 4.2.1) and SPSS 26.0 (IBM). Graphs were produced by Graphpad 8.0 and Adobe Illustrator CS6 (version 16.0.0). The details were described in Data S1.

## Results

### Betaine delayed muscle loss and improved mitochondrial function in aging

**Figure 1:**
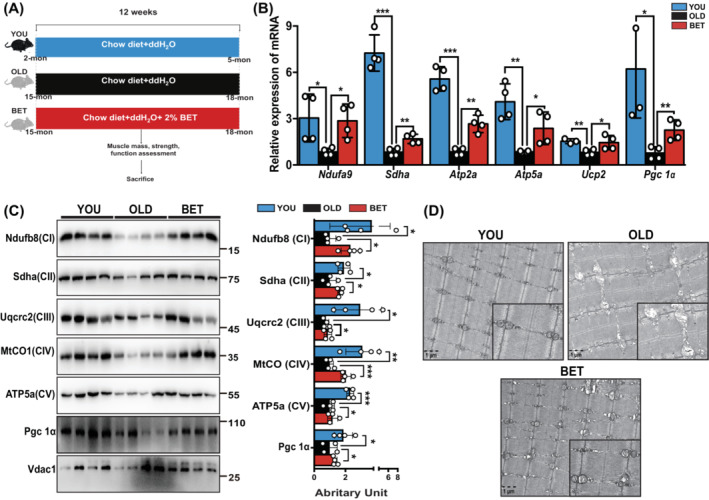
Betaine delayed muscle loss and improved mitochondrial function in aging. (A) Mice were divided into YOU (2 months), OLD (15 months), and BET (15 months) groups and raised for 12 weeks. The YOU and OLD groups received distilled water, while the BET group received distilled water with 2% (*w*/*v*) betaine. *N* = 6/group. (B) The qPCR experiment showed that betaine upregulated mRNA expressions of mitochondrial ETC molecules (*Sdha*, *Uqcrc2*, *MtCO*, and *ATP5a*) and biogenesis marker (*PGC‐1α*). Mean ± SD, *n* = 4/group. (C) Representative images of WB and quantification showed that betaine treatment preserved the expression of mitochondrial ETC‐related proteins (Ndufb8, Sdha, Uqcrc2, MtCO, and ATP5a). Mean ± SD, *n* = 4/group. (D) Representative TEM images demonstrated the subcellular structure of the sarcomere and mitochondrial morphology among the three groups. Betaine alleviated aging‐induced wasted mitochondrial vacuoles with better arrangement in the sarcomere. *N* = 4/group. *P < 0.05; **P < 0.01; ***P < 0.001.

To investigate the impact of betaine on muscle loss, mice of varying ages were raised for 12 weeks. The flow chart outlining the experimental procedures is depicted in Figure 1A. Aging could induce a decline in lean mass and an increase in fat mass (Figure S1A, B). Betaine demonstrated protective effects against the age‐related adverse body composition, and maintained muscle strength (BET vs. OLD: 74.8 ± 3.3 N/kg·BW vs. 63.7 ± 8.4 N/kg·BW, *P* = 0.010, Figure S1C). In the running test, old mice covered less distance in the given time compared with young mice. Following betaine treatment, aging mice were able to cover a longer distance in the running test (BET vs. OLD: 238.2 ± 6.7 m vs. 200.6 ± 20.5 m, *P* = 0.013, Figure S1D). H&E staining revealed that betaine treatment could mitigate age‐induced nucleus centralization and preserve muscle cross‐sectional area (Figure S1E, F), suggesting a potential protective role of betaine against age‐related muscle atrophy.

Age‐related muscle loss can be attributed in part to impaired mitochondrial function (respiration, biogenesis, and morphology).^21^ Significant decreases in the expression of mitochondrial ETC components (Sdha, Uqcrc2, MtCO, and ATP5a) and the biogenesis marker (PGC‐1α) at mRNA and protein levels were observed during the aging process, suggesting a deterioration in muscle metabolism (Figure 1B, C). The sarcomeres, as the fundamental units of muscle contraction, exhibited a higher presence of wasted mitochondrial vacuoles, along with disorganized dark and light bands in OLD group, indicating impairments in muscle function and structure during aging (Figure 1D). Betaine treatment was observed to partially reverse these age‐related effects on mitochondrial function in skeletal muscle.

### Betaine affected the pathways related to mitochondrial function and downregulated *Mss51* gene expression in aging skeletal muscle

**Figure 2:**
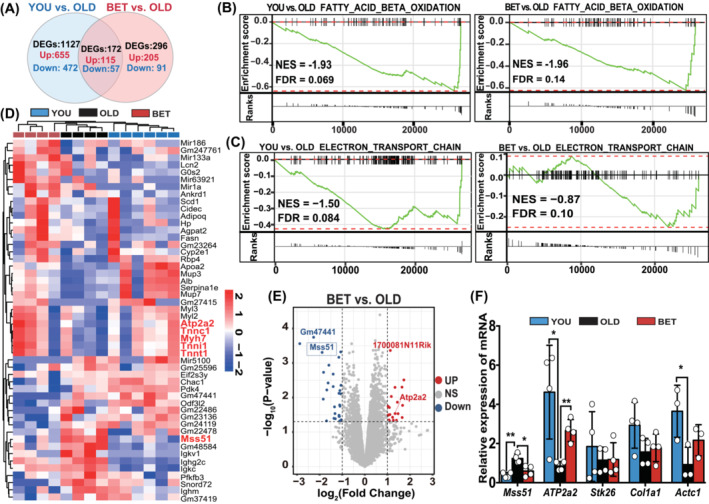
Betaine affected the pathways related to mitochondrial function and downregulated *Mss51* gene expression in aging skeletal muscle. (A) RNA sequencing results were obtained from the gastrocnemius. Compared with the OLD group, there were 1299 and 468 differentially expressed genes in the YOU and BET groups, respectively. *N* = 4–6/group. (B, C) GSEA results showed that pathways related to fatty acid beta‐oxidation and ETC were more enriched in the YOU and BET groups compared with the OLD group. *N* = 4–6/group. (D, E) The heatmap and volcano plot illustrated that several genes involved in skeletal muscle structure (*Tnnt1* and *Myh7*) and mitochondrial metabolism (*Atp2a2*) were upregulated in the BET and YOU groups, along with Mss51 downregulation. *N* = 4–6/group. (F) The qPCR results displayed that betaine negatively regulated the mRNA expression of *Mss51*, which could be aging‐induced. Mean ± SD, *n* = 4/group. *P < 0.05; **P < 0.01; ***P < 0.001.

To investigate the underlying mechanism of betaine on mitochondrial function improvement, RNA sequencing of mice gastrocnemius muscle was conducted. Comparing with the OLD group, 529 upregulated genes and 770 downregulated genes in the YOU group were identified, as well as 320 upregulated genes and 148 downregulated genes in the BET group (Figure 2A). Additionally, 172 genes shared similar expression patterns in both the YOU and BET groups, as depicted in the Venn diagram (Figure 2A).

Gene set enrichment analysis (GSEA) results revealed that aging significantly reduced the activity of the fatty acid beta‐oxidation pathway in the OLD group compared with both the YOU (NES = −1.93, FDR = 0.069) and BET (NES = −1.96, FDR = 0.14) groups (Figure 2B). Aging also inhibited the electron transport chain (ETC) activity in the OLD group relative to both the YOU (NES = −1.50, FDR = 0.084) and BET (NES = −0.87, FDR = 0.10) groups (Figure 2C). Similarly, the Gene Ontology (GO) enrichment analysis also indicated a higher enrichment of genes related to electron carrier activity and fatty acid beta‐oxidation in the YOU or BET groups (Figure S2A, B). These findings suggest that betaine treatment influences regulators associated with mitochondrial ETC function and skeletal muscle energy metabolism during aging.

To explore the potential target, the differential gene expression analysis was performed. The heatmap depicted the top 50 notably differential expressed genes. Among them, several genes were related to skeletal muscle structure (*Tnnt1* and *Myh7*) and mitochondrial metabolism (*Atp2a2*) in both the BET and YOU groups (Figure 2D). Furthermore, GSEA revealed these top genes could cluster into pathways related to muscle system processes in the YOU and BET groups (Figure S2C, NES = −0.94 to −0.79, FDR = 0.10). Besides, the gene expression of mitochondrial COX1 mRNA‐specific translational activator, *Mss51*, was consistently downregulated in the BET and YOU groups. Consistent with the heatmap, the volcano plot demonstrated *Mss51* was significantly downregulated in both the BET (Figure 2E) and YOU (Figure S2D) groups. Mss51 has been reported as a mitochondrial translation regulator.^22^, ^23^ These results indicated *Mss51* could be age‐induced in skeletal muscle and could be a potential target for betaine regulation. The further validation was derived from qRT‐PCR after excluding unknown, muscle non‐expressed, or mitochondria‐unrelated genes (Figure 2F).

### Betaine attenuated the Mss51‐mediated impairment in mitochondrial respiration

**Figure 3:**
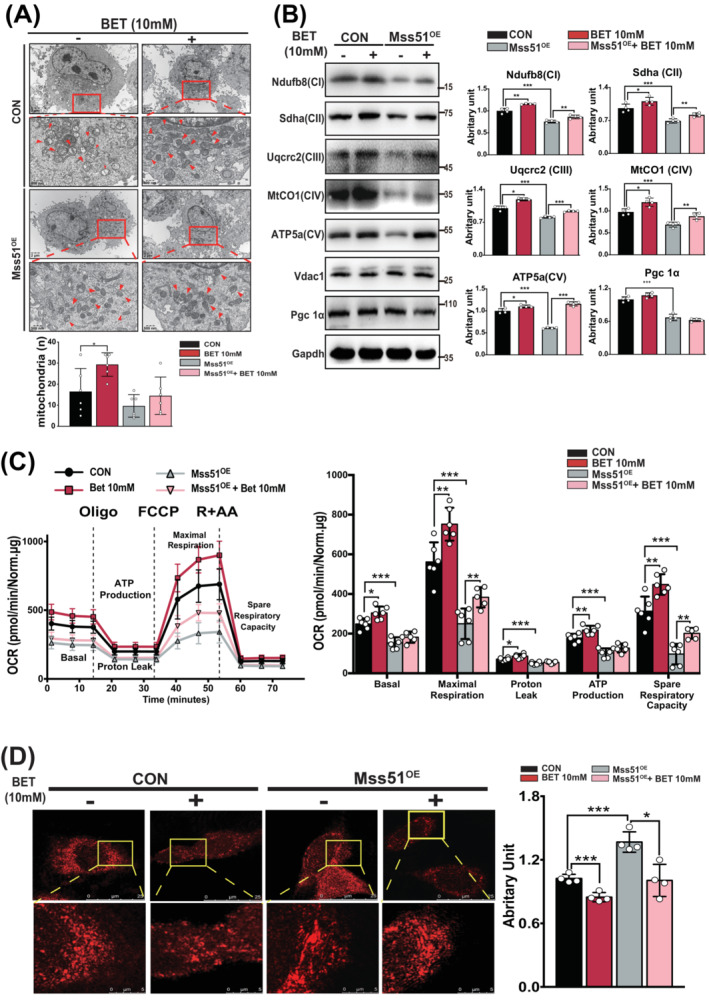
Betaine attenuated the Mss51‐mediated impairment in mitochondrial respiration. (A) Representative TEM images demonstrated that Mss51 induced an overall reduction in mitochondrial quantity in C2C12 cells, which could not be reversed by betaine. *N* = 4/group. (B) Presentative images of WB displayed betaine restored the inhibition in the mitochondrial ETC‐related protein expressions. Mean ± SD, *n* = 4/group. (C) Seahorse experiment demonstrated Mss51‐mediated suppression in the mitochondrial respiration could be attenuated by the betaine treatment. Mean ± SD, *n* = 5–6. (D) Presentative graphs of confocal microscopy illustrated betaine treatment decreased the accumulation of the mitochondrial superoxide elevated by the Mss51. The bottom panels showed larger regions highlighted in the yellow frames. Mean ± SD, *n* = 4/group. *P < 0.05; **P < 0.01; ***P < 0.001.

Even though Mss51 has been reported important to the mitochondrial function in yeast, the effects of Mss51 deletion on cellular metabolism, mitochondrial respiration, and quality control in skeletal muscle are conflicted.^22^, ^23^ To further investigate betaine effects on the Mss51, C2C12 cells with stable overexpression of FLAG‐tagged Mss51 were created (Figure S3A, B). Both control (CON) and Mss51‐overexpressing cells (Mss51^OE^) were treated with betaine. Results from TEM and WB confirmed that Mss51 overexpression reduced overall mitochondrial quantity in cells (Figure 3A) and suppressed the expression of proteins related to mitochondrial ETC and biogenesis (Figure 3B), suggesting impaired mitochondrial respiration and cellular energy metabolism. Betaine treatment only reversed Mss51's inhibition on mitochondrial ETC‐related protein expression (Figure 3B) but not the decline in mitochondrial numbers (Figure 3A). The consistent expression of PGC1α in Mss51^OE^ cells with or without betaine treatment further confirmed that betaine could not counteract the deterioration of Mss51‐induced mitochondrial biogenesis (Figure 3B).

We inferred that betaine's regulation of *Mss51* would have a greater impact on the mitochondrial respiration than on biogenesis. To test this, cellular oxygen consumption rate (OCR) of C2C12 cells was assessed by a Seahorse XFe96 analyser (Figure 3C). Compared with CON cells, Mss51^OE^ cells showed decreased rates of basal, maximal respiration, proton leak, ATP production, and spare respiratory capacity (Figure 3C). Betaine treatment reversed the decline in basal, maximal respiration, and spare respiratory capacity in Mss51^OE^ cells and had potential trend to increase basal respiration, proton leak, and ATP production (Figure 3C). The decrease in cellular OCR would lead to an accumulation of mitochondrial superoxide.^24^ Confocal microscopy images revealed higher levels of mitochondrial superoxide in Mss51^OE^ cells, which could be reduced by betaine treatment (Figure 3D, *P* = 0.001). These results indicated betaine could profoundly attenuate the Mss51‐mediated suppression in mitochondrial respiration and improve muscle energy metabolism.

### Betaine attenuated Yy1 age‐related decline and Yy1 transcriptionally downregulated *Mss51* transcription

**Figure 4:**
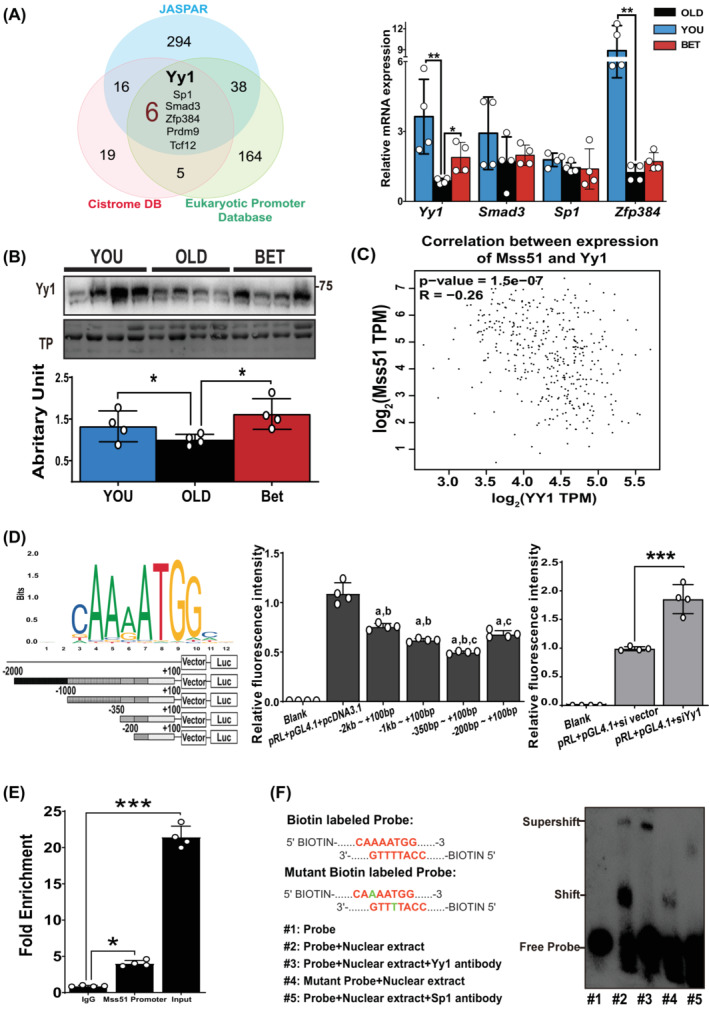
Betaine attenuated Yy1 age‐related decline and Yy1 transcriptionally downregulated the Mss51 transcription. (A) Six potential candidates were figured out by utilizing three databases. *Yy1* mRNA expressions were significantly higher in YOU and BET group. Mean ± SD, *n* = 4/group. (B) WB revealed that Yy1 protein level declined with aging and could be preserved by betaine treatment. Mean ± SD, *n* = 4/group. (C) GEPIA2 database indicates a negative correlation between the expression Yy1 and Mss51. (D) The full‐length promoter (T‐2000) and a series of truncated promoter (T‐1000, T‐350, and T‐200) into pGL4.10 luciferase reporter plasmids were co‐transfected with Yy1 plasmid in C2C12 cells. Relative fluorescence intensity (RFI) from Luciferase assay was compared, showing the downregulation of Yy1 on *Mss51* transcription. ^a^Significant different RFI compared with vector group. ^b^Significant difference by post‐hoc analysis for comparison among T‐2000, T‐1000, T‐350, and T‐200 groups. ^**c**^Significant different RFI between T‐350 and T‐200 groups. Mean ± SD, *n* = 4/group. (E) ChIP results demonstrated that Yy1 could bind to the region (−350 bp to +100 bp) of the *Mss51* promoter. Mean ± SD, *n* = 4/group. (F) EMSA showed that Yy1 could specifically bind to the *Mss51* promoter region with the sequence (5′ … CAAAATGG … 3′). *P < 0.05; **P < 0.01; ***P < 0.001.

Based on the downregulation of Mss51 function from betaine, potential transcriptional regulations related to betaine's impact on *Mss51* gene expression were explored. Using JASPAR, Cistrome, and the Eukaryotic Promoter Database, six putative transcript factors (Yy1, Sp1, Smad3, Zfp384, Prdm9, and Tcf12) were identified (Figure 4A). Two of these candidates (Prdm9 and Tcf12) were ruled out due to their either negligible or extremely low mRNA expression levels in muscle tissue. Among the remaining candidates, only Yy1 consistently and significantly higher expressed in YOU and BET groups compared with the OLD group (Figure 4A). WB also revealed Yy1 expression declined with aging and could be preserved by betaine treatment (Figure 4B).

The GEPIA2 database demonstrated the negative correlation between YY1 and Mss51 expression (Figure 4C). To validate roles of Yy1 in downregulating *Mss51* expression, the dual luciferase, ChIP, and EMSA were performed. Putative binding sites of *Mss51* promoter regions were generated based on the JASPAR database (Figure 4D) and co‐overexpressed with Yy1 plasmid in the C2C12 cells. The −350 to +100 bp region upstream of the *Mss51* transcription start site (TSS) showed the most significant decrease (~50%) in *Mss51* promoter activity (Figure 4D), while knocking‐down of Yy1 led to approximately 90% increase in the activity (Figure 4D). These results suggested the role of Yy1 in suppressing the *Mss51* transcription. Subsequently, ChIP used primer design from the site of –256 bp to −248 bp further confirmed *Mss51* promoter could interact with Yy1 (Figure 4E), and EMSA demonstrated specific binding of Yy1 to the sequence (5′ … CAAAATGG … 3′) (Figure 4F).

### Yy1 is of necessity for betaine improving Mss51‐induced mitochondrial respiration attrition

**Figure 5:**
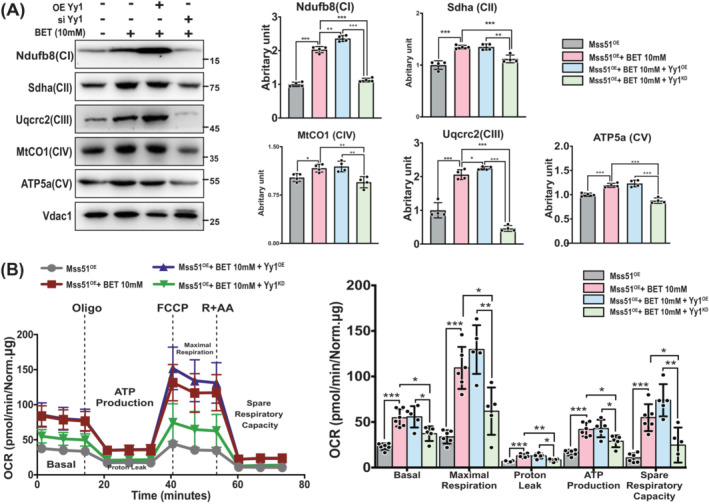
Yy1 involved in the improvement of betaine for the mitochondrial respiration and cellular energy metabolism in C2C12 cells. (A) Representative images of the WB showed that Yy1 was required for betaine to improve mitochondrial respiration. Mean ± SD, *n* = 4/group. (B) Seahorse experiments demonstrated that Yy1 was required for betaine to regulate C2C12 cellular energy metabolism. Mean ± SD, *n* = 4/group. *P* < 0.05; **P < 0.01; ***P < 0.001.

To assess the role of Yy1 involving improvement of betaine on Mss51‐induced mitochondrial respiration impairment, Yy1 were overexpressed or knocked down in the Mss51^OE^ C2C12 cells for further exploration (Figure S3C). Compared with Mss51^OE^ C2C12 cells with betaine treatment, overexpression of Yy1 alongside Mss51^OE^ cells with betaine treatment showed increased mitochondrial respiration protein expression. Knocking‐down Yy1 in Mss51^OE^ cells reduced the positive impact of betaine on mitochondrial ETC proteins expression (Figure 5A). Seahorse experiments further displayed Mss51^OE^ cells with Yy1 knocked down had the lowest OCRs at basal, maximal, and spare time, as well as less proton leak and ATP production among groups (Figure 5B). Conversely, overexpressing Yy1 in Mss51^OE^ cells tended to have the highest oxygen consumption rate among groups (Figure 5B). These results implied the necessity of Yy1 for betaine improving Mss51‐induced mitochondrial respiration attrition.

**Figure 6:**
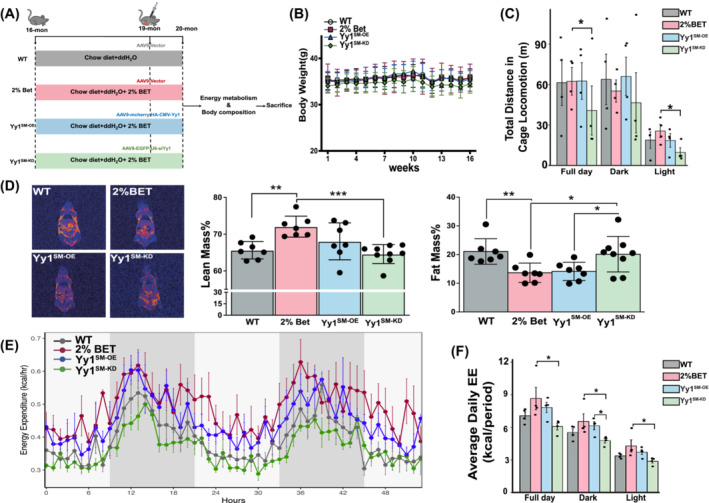
Yy1 involved in the improvement of betaine for the muscle loss and energy metabolism attrition. (A) The flow chat of the skeletal specific gain‐loss function of the Yy1 by the AAV transfection. Mice were divided into the following four groups: WT, 2%BET, Yy1^SM‐OE^, Yy1^SM‐KD^, *n* = 10/group. (B) Changes in body weight among the four groups during the intervention. Mean ± SD, *n* = 7–9/group. (C) Knocking down Yy1 in skeletal muscle diminished the favourable effects of betaine on physical performance (distance covered in the cage). Mean ± SD, *n* = 4/group. (D) Downregulation in Yy1 reduced the lean mass% and increased the fat mass%. Mean ± SD, *n* = 7–9/group. (E, F) Depletion of the Yy1 impaired the improvement of betaine on the daily energy expenditure. Mean ± SD, *n* = 4/group. *P* < 0.05; **P < 0.01; ***P < 0.001.

The *in vitro* findings presented above indicated the essential role of Yy1 in mediating the benefit effect of betaine on mitochondrial respiration. To further corroborate these observations *in vivo*, 16‐month‐old mice were allocated into different groups (WT, BET, YY1^SM‐OE^, and YY1^SM‐KD^). Expect for the WT group, the other three groups were provided with distilled water containing 2% betaine (*w*/*v*). The details of the interventions are outlined in Figure 6A. AAVs carrying plasmids for Yy1 overexpression or depletion were injected into the TA muscle after 12 weeks, while the other two groups received injections of an equivalent volume of 0.9% saline. The efficiencies of the AAV‐induced Yy1 overexpression (~145 folds) and knocking‐down (decrease 82%) *in vivo* were presented in Figure S3D, E. After intervention, the body weight was comparable among the four groups (Figure 6B). The deletion of Yy1 attenuated the favourable effects of betaine on running distance (Figure 6C, *P* = 0.046) and body composition (Figure 6D). Furthermore, the YY1^SM‐KD^ group exhibited a reduction in energy expenditure compared with the BET group (Figure 6E, F, *P* = 0.015).

**Figure 7:**
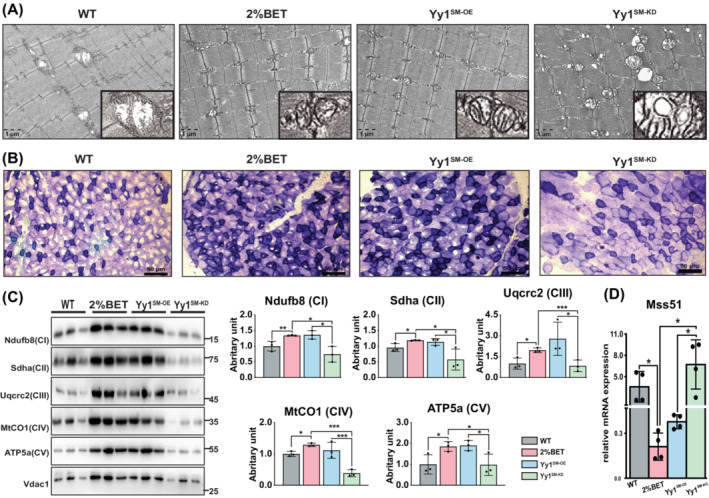
Betaine attenuated the aging‐induced inhibition of mitochondrial respiration is dependent of Yy1. (A) Representative images from TEM exhibited the attenuation of the betaine effects on the mitochondrial and sarcomere morphological levels after the loss of Yy1, *n* = 4/group. (B) The SDH staining illustrated loss of Yy1 impacted the activation of the betaine on the mitochondrial respiration, *n* = 4/group. (C) Representative images of WB and the quantification demonstrate that Yy1 is required for the enhancement of betaine on mitochondrial respiration. Mean ± SD, *n* = 3/group. (D) The qPCR result illustrated betaine suppression on the *Mss51* expression was dependent of the transcript factor Yy1. Mean ± SD, *n* = 4/group. *P* < 0.05; **P < 0.01; ***P < 0.001.

Mitochondrial respiration and sarcomere structure are important for maintenance of muscle strength and function. The results from TEM illustrated in contrast with YY1^SM‐KD^ group; there were less wasted mitochondrial vacuolization and better arranged sarcomere in both the BET and YY1^SM‐OE^ groups (Figure 7A). SDH staining was performed to assess mitochondrial respiration activity, which indicated a decline of mitochondrial respiration in the YY1^SM‐KD^ group exhibited (Figure 7B). Similar results were observed at the translational level for mitochondrial‐related proteins (Figure 7C). Finally, the transcriptional expression of *Mss51* was assessed via qRT‐PCR. Consistently with the result in luciferase assay, the results indicated that Yy1 suppressed the *Mss51* gene expression *in vivo*, and deficiency in skeletal muscle significantly diminished the betaine‐mediated downregulation of *Mss51* (Figure 7D). Translational analysis of mitochondrial‐related proteins (Figure 7C) and qRT‐PCR of *Mss51* expression (Figure 7D) further supported role of Yy1 in mediating betaine's effects on improving mitochondrial respiration and skeletal muscle energy metabolism to delay age‐related muscle loss.

## Discussion

In our study, it is suggested that betaine supplementation may alleviate age‐related muscle loss by reduce *Mss51* mRNA expression via the transcription factor Yy1. The modulation leaded to enhanced mitochondrial respiration, increased energy expenditure and running distance, which were closely associated with the preservation of muscle mass, strength, and physical performance in advanced aging.

Mitochondria perform diverse yet interconnected functions, acting as the hub of energy metabolism.^25^ They are also crucial for nutrient turnover, ATP production, and the synthesis of various intermediates.^26^ By participating in essential metabolic pathways, mitochondria are integrated into intracellular signalling networks that regulate various cellular functions. It is not surprising that mitochondrial dysfunction has emerged as a key factor in aging and numerous diseases, including the muscle degeneration,^27^ cancer, and other disease.^28^ Consistent with previous studies, we observed several characteristic phenotypes of mitochondrial dysfunction in aging skeletal muscle, such as decreased expression of mitochondrial respiration proteins, a reduced number of mitochondria, and the accumulation of dysfunctional mitochondrial vacuoles (Figure 1D). These results suggest that age‐related mitochondrial dysfunction worsens energy metabolism and physical performance in aging skeletal muscle. Therefore, strategies targeting mitochondria are urgently needed to address the growing burden from aging populations worldwide as early as possible.

In the current study, we used mice of different ages to investigate age‐related declines in muscle health and mitochondrial function, particularly mitochondrial respiration. We found that betaine could alleviate age‐related declines in mitochondrial function, including upregulating the expression of mitochondrial ETC‐related proteins and improving age‐induced histopathological changes in mitochondrial structure (Figure 1). While previous studies on betaine's effects on aging skeletal muscle mitochondria are limited, its ability to regulate mitochondrial function has been widely reported in liver tissue. For example, betaine could promote mitochondrial dynamics by activating MFN expression^29^; betaine could inhibit fatty acid synthesis to regulate lipid metabolism, ameliorating symptoms of NAFLD and NASH.^30^ Our discovery of betaine's benefit impacts on aging skeletal muscle mitochondrial respiration provides novel evidence for its potential application in delaying age‐related skeletal muscle loss. These results are promising, given the easy availability and adherence of betaine dietary supplementation.

Numerous genes are essential for coordinating normal mitochondrial respiration function. Compared with the OLD group, there was a significant downregulation of *Mss51* in the BET and YOU groups. Bioinformatics analysis, along with validation through qRT‐PCR, revealed an age‐induced increase in *Mss51* expression (Figure 2D–F, Figure S2D), which could be reduced after betaine treatment. From our GSEA results, we found along with the downregulation of the *Mss51*, betaine activated the fatty acid beta‐oxidation and ETC activity (Figure 2B, C, Figure S2B). Meanwhile, betaine treatment was correlated with the pathway of muscle system process (Figure S2C). While Mss51 is crucial for the assembly of COX1 in the mitochondrial ETC in *Saccharomyces cerevisiae*, ^13^ its function in mice or humans differs due to gene heterogeneity.^31^ Results from the study of Yoshioka et al. revealed that the Mss51 was a skeletal muscle‐specific regulator essential for the cellular metabolism.^22^ In opposite, Wagner et al. demonstrated that Mss51 could be stress‐induced and negatively regulated the mitochondria function.^32^ The deletion of Mss51 would enhance the glucose homeostasis and mitochondrial respiration, increase the exercise endurance, and ameliorate the muscle histopathology.^23^ Notably, a recent human study demonstrated Mss51 is associated with the incidence of type 2 diabetes, indicating its adverse role in regulating the energy metabolism.^17^ In accordance with the results of Wagner et al., we confirmed the adverse effects of Mss51 on the mitochondrial biogenesis and the mitochondrial energy metabolism (Figure 3). The differences in underlying mechanisms and animal models may account for these conflicting results.

By supplementing C2C12 cells with betaine, we confirmed that betaine could significantly rescue the inhibition of mitochondrial respiration caused by Mss51 (Figure 3). Previous studies indicate that Mss51 function can be transcriptionally regulated,^16^ prompting us to consider that the underlying mechanism of betaine in regulating Mss51 function may be related to transcription factors. Further exploration identified six candidates that could transcriptionally regulate the expression of Mss51. Through luciferase assays, ChIP, and EMSA, it was confirmed that the transcription factor Yy1 specifically and negatively regulates *Mss51* expression (Figure 4D–F). Yy1 is well‐documented as a crucial regulator of metabolic homeostasis.^33^ ^,^ ^34^ Previous literature has shown that the deletion of Yy1 significantly inhibits the expression of mitochondrial ETC‐related molecules, thereby compromising mitochondrial OXPHOS and impairing muscle function.^35^ Additionally, Yy1 can inhibit TGF‐β signalling in the nucleus.^36^ TGF‐β signalling is known to regulate Mss51 activation upstream,^16^ providing an explanation for how upregulation of Yy1 by betaine could inhibit *Mss51* expression and enhance mitochondrial respiration.

Our results demonstrate that the loss of Yy1 diminishes the beneficial effects of betaine on mitochondrial respiration and oxygen consumption rate in C2C12 cells (Figure 5). As such, we anticipated that betaine regulation on the mitochondrial respiration via suppressing the Mss51 should be dependent on the Yy1. Therefore, we hypothesized that betaine's regulation of mitochondrial respiration by suppressing Mss51 is dependent on Yy1. This hypothesis was supported by our *in vivo* study using 16‐month‐old mice. Through gain‐loss‐function experiments of Yy1 in the TA muscle, we found that Yy1 is necessary for betaine's regulation of mitochondrial respiration. The loss of Yy1 resulted in an increase in fat mass percentage and impaired exercise ability (Figure 6). Mechanistically, we observed a significant upregulation of *Mss51* expression in the TA muscle upon depletion of Yy1 (Figure 7), further illustrating the dependence of betaine's inhibition of *Mss51* on Yy1. Given our previous study demonstrating that betaine activates the mTORC1 signalling pathway,^9^ and Puigserver et al. confirming that mTORC1 controls mitochondrial OXPHOS through the Yy1‐PGC1α complex,^33^ we suspected that the mTORC1 signalling pathway may link betaine and Yy1 in regulating *Mss51* expression. Further studies are warranted to explore this mechanism in the future.

Interestingly, the overexpression of Yy1 did not lead to significant improvements in mitochondria or skeletal muscle function *in vivo* or *in vitro*, as expected (Figures 5, 6, 7). We speculated the absence of Yy1 could greatly compromise energy metabolism and other essential biological processes through intricate transcriptional regulatory pathways, including signalling activation, cell proliferation, and differentiation.^37^ All these compromised processes would significantly reduce the beneficial effects of betaine. It is possible that the impact of betaine on mitochondrial improvement may have already reached its peak, thereby making the improvement from Yy1 overexpression in mitochondrial respiration less noticeable. Additionally, it remains unknown whether there is a longer time window for the AAV‐transfection of Yy1 to exert effects on betaine regulation, which may require further exploration in the future.

In conclusion, our study demonstrates that dietary supplementation with betaine can alleviate age‐related suppression of mitochondrial respiration and mitigate skeletal muscle loss. Our findings suggest that the application of betaine supplementation, either alone or in combination with other interventions, holds promise for clinical translation in delaying muscle loss, improving energy metabolism in aging skeletal muscle, and potentially extending health span.

## Conclusion

In conclusion, our study demonstrates that dietary supplementation with betaine can alleviate age‐related suppression of mitochondrial respiration and mitigate skeletal muscle loss. Our findings suggest that the application of betaine supplementation, either alone or in combination with other interventions, holds promise for clinical translation in delaying muscle loss, improving energy metabolism in aging skeletal muscle, and potentially extending health span.

## Conflict of interest

The authors declare no conflicts of interest.

## Supporting information


**Data S1.** Supporting Information.

**Figure S1.** Betaine alleviated skeletal muscle loss (mass, strength, physical performance) during the aging process. (A‐B) Aging‐induced adverse alterations in body composition could be alleviated by betaine treatment. Mean and SD, *n* = 5–6/group. Betaine partially prevented against (A) decrease in muscle mass and (B) increase in fat mass with age. (C) Betaine alleviated age‐related decline in muscle strength. Mean and SD, *n* = 6/group. (D) Betaine preserved the ability of muscle performance (running distance, response time to electronic shock) during aging. Mean and SD, *n* = 4 per group. (E) Representative figures of H&E staining depict that betaine reduced the centralization of muscle nuclei (yellow arrows) and (F) protected against age‐related atrophy in the skeletal muscle cross‐sectional area. *n* = 4/per group. *: *P* < 0.05; **: *P* < 0.01; ***: *P* < 0.001.
**Figure S2.** Bioinformatic analysis of pathways altered during aging and betaine treatment in skeletal muscle. (A‐B) GO analysis indicated a higher enrichment of genes related to electron carrier activity and fatty acid beta‐oxidation in the YOU or BET groups. *n* = 4–6/group. (C) The top 50 genes among the three groups were employed for GSEA. Pathways clustered into the muscle system process in the YOU and BET groups. *n* = 4–6/group. (D) The volcano plot demonstrates that compared with the OLD group, *Mss51* mRNA expression was significantly lower in the YOU group. n = 4–6/group.
**Figure S3.** (A) C2C12 cells stably overexpressing Mss51 were established. The efficiency of lentivirus vector transduction into the C2C12 cells was demonstrated by GFP expression. (B) Western blotting was performed to evaluate the efficiency of Mss51 transfection in C2C12 cells. (C) Western blotting was performed to evaluate the efficiency of Yy1 OE (~50%) and KD (~80%) in C2C12 cells. (D‐E) Western blotting was performed to evaluate the efficiency of AAV‐mediated Yy1 OE (~145‐folds) and KD (~82%) *in vivo.*.

**Table S1.** Primer sequences used to amplify DNA.
**Table S2.** Plasmid sequences of lentiviral vectors of Mss51.
**Table S3.** Yy1 plasmid sequences for Dual‐luciferase report assay.
**Table S4.** Mss51 promoter plasmid sequences for Dual‐luciferase report assay.
**Table S5.** Probe design for EMSA.

**Data S2.** Supporting Information.

## Data Availability

Datasets generated during the current study are available from the corresponding author on reasonable request.

## References

[1] Wiedmer P , Jung T , Castro JP , Pomatto LCD , Sun PY , Davies KJA , et al. Sarcopenia ‐ molecular mechanisms and open questions. Ageing Res Rev 2021;65:101200. 33130247 10.1016/j.arr.2020.101200

[2] Petermann‐Rocha F , Balntzi V , Gray SR , Lara J , Ho FK , Pell JP , et al. Global prevalence of sarcopenia and severe sarcopenia: a systematic review and meta‐analysis. J Cachexia Sarcopenia Muscle 2022;13:86–99. 34816624 10.1002/jcsm.12783 PMC8818604

[3] Lopez‐Otin C , Blasco MA , Partridge L , Serrano M , Kroemer G . Hallmarks of aging: an expanding universe. Cell 2023;186:243–278. 36599349 10.1016/j.cell.2022.11.001

[4] Ubaida‐Mohien C , Lyashkov A , Gonzalez‐Freire M , Tharakan R , Shardell M , Moaddel R , et al. Discovery proteomics in aging human skeletal muscle finds change in spliceosome, immunity, proteostasis and mitochondria. Elife 2019;8. 10.7554/eLife.49874 PMC6810669 31642809

[5] Marzetti E , Calvani R , Cesari M , Buford TW , Lorenzi M , Behnke BJ , et al. Mitochondrial dysfunction and sarcopenia of aging: from signaling pathways to clinical trials. Int J Biochem Cell Biol 2013;45:2288–2301. 23845738 10.1016/j.biocel.2013.06.024 PMC3759621

[6] Coen PM , Jubrias SA , Distefano G , Amati F , Mackey DC , Glynn NW , et al. Skeletal muscle mitochondrial energetics are associated with maximal aerobic capacity and walking speed in older adults. J Gerontol A Biol Sci Med Sci 2013;68:447–455. 23051977 10.1093/gerona/gls196 PMC3593613

[7] Craig SA . Betaine in human nutrition. Am J Clin Nutr 2004;80:539–549. 15321791 10.1093/ajcn/80.3.539

[8] Long JA , Zhong RH , Chen S , Wang F , Luo Y , Lu XT , et al. Dietary betaine intake is associated with skeletal muscle mass change over three years in middle‐aged adults: the Guangzhou Nutrition and Health Study. Br J Nutr 2020;1‐21. 10.1017/S0007114520002433 32616104

[9] Chen S , Lu XT , He TT , Yishake D , Tan XY , Hou MJ , et al. Betaine delayed muscle loss by attenuating samtor complex inhibition for mTORC1 signaling via increasing SAM level. Mol Nutr Food Res 2021;65:e2100157. 34061446 10.1002/mnfr.202100157

[10] Lee I . Betaine is a positive regulator of mitochondrial respiration. Biochem Biophys Res Commun 2015;456:621–625. 25498545 10.1016/j.bbrc.2014.12.005

[11] Heidari R , Niknahad H , Sadeghi A , Mohammadi H , Ghanbarinejad V , Ommati MM , et al. Betaine treatment protects liver through regulating mitochondrial function and counteracting oxidative stress in acute and chronic animal models of hepatic injury. Biomed Pharmacother 2018;103:75–86. 29635131 10.1016/j.biopha.2018.04.010

[12] Zhang L , Qi Y , ALuo Z , Liu S , Zhang Z , Zhou L . Betaine increases mitochondrial content and improves hepatic lipid metabolism. Food Funct 2019;10:216–223. 30534761 10.1039/c8fo02004c

[13] Soto IC , Fontanesi F , Myers RS , Hamel P , Barrientos A . A heme‐sensing mechanism in the translational regulation of mitochondrial cytochrome c oxidase biogenesis. Cell Metab 2012;16:801–813. 23217259 10.1016/j.cmet.2012.10.018 PMC3523284

[14] Rovira Gonzalez YI , Moyer AL , LeTexier NJ , Bratti AD , Feng S , Pena V , et al. Mss51 deletion increases endurance and ameliorates histopathology in the mdx mouse model of Duchenne muscular dystrophy. FASEB J 2021;35:e21276. 33423297 10.1096/fj.202002106RR

[15] Yoshioka K , Fujita R , Seko D , Suematsu T , Miura S , Ono Y . Distinct roles of Zmynd17 and PGC1alpha in mitochondrial quality control and biogenesis in skeletal muscle. Front Cell Dev Biol 2019;7:330. 31921843 10.3389/fcell.2019.00330 PMC6915033

[16] Mousa MG , Vuppaladhadiam L , Kelly MO , Pietka T , Ek S , Shen KC , et al. Site‐1 protease inhibits mitochondrial respiration by controlling the TGF‐beta target gene Mss51. Cell Rep 2023;42:112336. 37002920 10.1016/j.celrep.2023.112336 PMC10544680

[17] Ali S , Ahmad K , Shaikh S , Chun HJ , Choi I , Lee EJ . Mss51 protein inhibition serves as a novel target for type 2 diabetes: a molecular docking and simulation study. J Biomol Struct Dyn 2023;1‐8:4862–4869. 10.1080/07391102.2023.2223652 37338036

[18] Mahmoud AM , Ali MM . Methyl donor micronutrients that modify DNA methylation and cancer outcome. Nutrients 2019;11:608. 30871166 10.3390/nu11030608 PMC6471069

[19] Millian NS , Garrow TA . Human betaine‐homocysteine methyltransferase is a zinc metalloenzyme. Arch Biochem Biophys 1998;356:93–98. 9681996 10.1006/abbi.1998.0757

[20] Cortopassi MD , Ramachandran D , Rubio WB , Hochbaum D , Sabatini BL , Banks AS . Analysis of thermogenesis experiments with CalR. Methods Mol Biol 2022;2448:43–72. 35167089 10.1007/978-1-0716-2087-8_3

[21] Penniman CM , Bhardwaj G , Nowers CJ , Brown CU , Junck TL , Boyer CK , et al. Loss of FoxOs in muscle increases strength and mitochondrial function during aging. J Cachexia Sarcopenia Muscle 2023;14:243–259. 36442857 10.1002/jcsm.13124 PMC9891940

[22] Yoshioka K , Fujita R , Seko D , Suematsu T , Miura S , Ono Y . Distinct roles of Zmynd17 and PGC1α in mitochondrial quality control and biogenesis in skeletal muscle. Front Cell Dev Biol 2019;7:330. 31921843 10.3389/fcell.2019.00330 PMC6915033

[23] Rovira Gonzalez YI , Moyer AL , LeTexier NJ , Bratti AD , Feng S , Sun C , et al. Mss51 deletion enhances muscle metabolism and glucose homeostasis in mice. JCI Insight 2019;4. 10.1172/jci.insight.122247 PMC6824300 31527314

[24] Donatienne dH , Danhier P , Northshield H , Isenborghs P , Jordan BF , Gallez B . A versatile EPR toolbox for the simultaneous measurement of oxygen consumption and superoxide production. Redox Biol 2021;40:101852. 33418140 10.1016/j.redox.2020.101852 PMC7804984

[25] Boengler K , Kosiol M , Mayr M , Schulz R , Rohrbach S . Mitochondria and ageing: role in heart, skeletal muscle and adipose tissue. J Cachexia Sarcopenia Muscle 2017;8:349–369. 28432755 10.1002/jcsm.12178 PMC5476857

[26] Protasi F , Pietrangelo L , Boncompagni S . Improper remodeling of organelles deputed to Ca(2+) handling and aerobic atp production underlies muscle dysfunction in ageing. Int J Mol Sci 2021;22:6195. 34201319 10.3390/ijms22126195 PMC8228829

[27] Abrigo J , Simon F , Cabrera D , Vilos C , Cabello‐Verrugio C . Mitochondrial dysfunction in skeletal muscle pathologies. Curr Protein Pept Sci 2019;20:536–546. 30947668 10.2174/1389203720666190402100902

[28] Qiu F , Yuan Y , Luo W , Gong YS , Zhang ZM , Liu ZM , et al. Asiatic acid alleviates ischemic myocardial injury in mice by modulating mitophagy‐ and glycophagy‐based energy metabolism. Acta Pharmacol Sin 2022;43:1395–1407. 34522006 10.1038/s41401-021-00763-9 PMC9160258

[29] Jung KM . Betaine enhances the cellular survival via mitochondrial fusion and fission factors, MFN2 and DRP1. Anim Cells Syst (Seoul) 2018;22:289–298. 30460110 10.1080/19768354.2018.1512523 PMC6171430

[30] Yang W , Huang L , Gao J , Wen S , Tai Y , Chen M , et al. Betaine attenuates chronic alcohol‐induced fatty liver by broadly regulating hepatic lipid metabolism. Mol Med Rep 2017;16:5225–5234. 28849079 10.3892/mmr.2017.7295 PMC5647077

[31] Baleva MV , Piunova UE , Chicherin IV , Krasavina DG , Levitskii SA , Kamenski PA . Yeast translational activator Mss51p and human ZMYND17 ‐ two proteins with a common origin, but different functions. Biochemistry (Mosc) 2021;86:1151–1161. 34565318 10.1134/S0006297921090108

[32] Moyer AL , Wagner KR . Mammalian Mss51 is a skeletal muscle‐specific gene modulating cellular metabolism. J Neuromuscul Dis 2015;2:371–385. 26634192 10.3233/JND-150119 PMC4664537

[33] Cunningham JT , Rodgers JT , Arlow DH , Vazquez F , Mootha VK , Puigserver P . mTOR controls mitochondrial oxidative function through a YY1‐PGC‐1alpha transcriptional complex. Nature 2007;450:736–740. 18046414 10.1038/nature06322

[34] Weintraub AS , Li CH , Zamudio AV , Sigova AA , Hannett NM , Day DS , et al. YY1 is a structural regulator of enhancer‐promoter loops. Cell 2017;171:e28. 10.1016/j.cell.2017.11.008 PMC5785279 29224777

[35] Li B , Wang J , Liao J , Wu M , Yuan X , Fang H , et al. YY1 promotes pancreatic cancer cell proliferation by enhancing mitochondrial respiration. Cancer Cell Int 2022;22:287. 36123703 10.1186/s12935-022-02712-w PMC9484254

[36] Gao P , Li L , Yang L , Gui D , Zhang J , Han J , et al. Yin Yang 1 protein ameliorates diabetic nephropathy pathology through transcriptional repression of TGFbeta1. Sci Transl Med 2019;11. 10.1126/scitranslmed.aaw2050 31534017

[37] Chen F , Zhou J , Li Y , Zhao Y , Yuan J , Cao Y , et al. YY1 regulates skeletal muscle regeneration through controlling metabolic reprogramming of satellite cells. EMBO J 2019;38. 10.15252/embj.201899727 PMC6518041 30979776

[38] von Haehling S , Coats AJS , Anker SD . Ethical guidelines for publishing in the *Journal of Cachexia, Sarcopenia and Muscle*: update 2021. J Cachexia Sarcopenia Muscle 2021;12:2259–2261. 34904399 10.1002/jcsm.12899 PMC8718061

