# Supplementary material for: Betaine delays age‐related muscle loss by mitigating Mss51‐induced impairment in mitochondrial respiration via Yin Yang1
Source: J Cachexia Sarcopenia Muscle. 2024 Aug 26;15(5):2104–17. doi: 10.1002/jcsm.13558 (PMC11446699; doi:10.1002/jcsm.13558)
Supplement: Supplementary file 1 — Data S1. Supporting Information. [file JCSM-15-2104-s001.docx]

**Supplemental File1**

**1.1 Histological analyses**

Gastrocnemius muscle was isolated for the histological analyses. The muscle samples were fixed by the 4% paraformaldehyde, then embed by the paraffin block. The histological sections (5-μm) were then staining by the hematoxylin and eosin (H&E). The cross-sectional area was assessed by the Image J software (version 1.52a, NIH, USA). For SDH staining, muscle was embedded by OCT gum and immediately stored immediately at -80℃ until sectioning. Frozen muscles were processed into 15 μm thick sections at a cryostat (Leica Biosysterms). Sections were stained with SDH dye as then processed as the manufacture introduction (Solarbio, Chnia).

**1.2 Transmitted electronic microscopy (TEM)**

The isolated gastrocnemius muscle was rapidly fixed in the 2.5 % glutaraldehyde (Servicebio, China)，and stored at 4℃ before photography. The TEM (JOEL, Japan) analyses were performed by the Regional tests & pathology center of KingMed Diagnostics.

**1.3 Construction and transfection of lentiviral vectors**

For stable transduction of Mss51 in C2C12 cell, the Mss51 lentivirus expression vector (pSLenti-EF1-EGFP-P2A-Puro-CMV-3xFLAG-WPRE) were constructed by the OBIO (Inc, Shanghai, China). Briefly, the lentiviral vector, along with the packaging plasmids, is transfected into HEK293T cells. The transfected HEK293T cells produce lentiviral particles that can be harvested from the cell culture supernatant for later experiments. The multiplicity of infection (MOI) was 50 in the current study. When the cells confluency reaches to 50%, the plasmid of pSLenti-EF1-EGFP-P2A-Puro-CMV-Mss51-3xFLAG-WPRE or empty vector were added into the medium. The puromycin-resistant (3 µg/ mL) cells were then established. The control group (CON) and Mss51-overexpressed C2C12 (Mss51^OE^) were then treated with or without betaine (0, 10mM). The sequences and gene IDs of the Mss51 were presented in the **Supplemental Table 2.**

**1.4 Seahorse oxygen consumption rates (OCR) analysis**

Cellular respiration was evaluated on a Seahorse XFe96 Analyzer (Aglient, USA) and the Seahorse Mitochondrial Stress Test kit were applied for the assessment of cellular respiration. C2C12 cells were seeded in the Seahorse XF96 plate at a cell density of 10000. Cells were cultured in the Seahorse XF-DMEM with 1mM pyruvate, 10mM glucose and 2mM glutamine and incubated in a CO_2_ free incubator at 37℃ for 1h. The OCRs were measured as the instruction of the Seahorse Mitochondrial Stress Test protocol (Aglient, USA). Basal OCR were measured first, then sequential addition of oligomycin (1μM), carbonyl cyanide p-trifluoro-methoxyphenyl hydrazine (FCCP, 1μM), rotenone and antimycin A (0.5 mΜ respectively) were applied for the following OCRs (ATP-linked respiration, maximal respiration, spare respiratory capacity) assessment. After completion of the assay, whole protein lysates for each well were quantified by BCA assay and total protein amounts were used for normalization of the data using the Wave Software (Aglient, USA).

**1.5 Luciferase reporter assay**

We defined Mss51 gene promoter as regions from −2000 bp upstream of the transcription start site (TSS) to +100 bp downstream of the TSS. The YY1 binding sites on the Mss51 promoter were predicted using the JASPAR database (https://jaspar.genereg.net/). Based on the predicted binding sites (-1628 ~ -1620, -579 ~ -571, -395 ~ -387, -256 ~ -248), we cloned the full-length promoter (T-2000) and a series of truncated promoter (T-1000, T-350, T-200) into pGL4.10 luciferase reporter plasmids (OBIO, Inc, Shanghai, China). The pCDNA3.1-Yy1 as well as empty vector plasmids were also purchased from OBIO (Inc, Shanghai, China). Ablation of Yy1 was conducted by transient transfection with siRNA duplex oligos, which were synthesized by RIOBO (Inc, Guangzhou, Chian). The sequence of the si-Yy1 was as follows: 5ʹ-CCAGAATGAAGCCAAGAAA-3ʹ.

The C2C12 reached to 85% confluency were transfected with 0.9 μg pGL4.10 basic vector or the pGL4.10 vector harboring the various Mss51 promoter regions using Lipofectamine^TM^3000 (Invitrogen) as the guideline. The Renilla luciferase reporter pRL-CMV plasmid (OBIO, Shanghai, China), 0.09 μg per well, was co-transfected as the internal control. To examine the relationship between YY1 and Mss51 promoter activity, various Mss51 promoter regions reporter plasmid was co-transfected with YY1 or vector plasmid. Dual-luciferase assay was performed using the Dual-luciferase Reporter Assay kit (Promega, USA). Luciferase activities were measured with an illuminometer and normalized to the Renilla activity value (Promega GLOMAX, version 1.9.0, USA). The sequences for the luciferase assay are presented in the **Supplemental Table 3-4.**

**1.6 Chromatin immunoprecipitation (ChIP)**

The ChIP was performed to examine the interaction between Yy1 and Mss51 promoter region (T-350, -256 ~ -248), according to the protocol provided with the Pierce Magnetic ChIP Kit (Thermo scientific, USA). Briefly, C2C12 cells at a number of 4×10^6^ were crosslinked by the 1% formaldehyde. Then the cell membrane and cytosol were lysed and digested and followed by the sonication at an intensity of 25% electronic pulses for 15 cycles. This lysis was incubated on ice at a 30-second interval. After centrifugation, the supernatant was applied for the immunoprecipitation. The supernatant was incubated either with antibodies specific for Yy1 (#46395, CST) or with normal mouse IgG mixing with the ChIP grade protein A/G magnetic beads. The IP elution and DNA elution were subsequently performed. And the qPCR detection was conducted to determine the interaction of the Yy1 and the Mss51 promoter region. The sequence for the ChIP was presented in the **Supplemental Table1.**

**1.7 Electrophoretic Mobility Shift Assay (EMSA)**

The EMSA was conducted according to the LightShift™ EMSA kit manual (Thermo Scientific™, USA). Briefly, C2C12 cells at 4×10^6^ were harvested, and the Nuclear and cytosolic fractions were conducted using Nuclear and Cytoplasmic Protein Extraction Kit (Beyotime, P0028) according to the instructions. The nuclear extract was prepared from the lysate. The DNA probes containing the target DNA sequence of interest were design and the sequences were listed in the **Supplemental Table 5**. The binding reaction was then performed by the manual of the kit. Then, the binding reaction mixture was loaded on a non-denaturing polyacrylamide gel in 0.5x TBE buffer. Antibodies specific for Yy1 (#46395, CST) and Sp1 (ab227383, Abcam) were employed for supershift.

**1.8 Statistical analysis**

Statistical analyses were performed with SPSS 26.0 (IBM) and R software (version 4.2.1). One way ANOVA, followed by Dunnett’T3 post hoc test for comparison between two groups. Significance was defined as a *P* value of 0.05. For the analysis of the RNA-Seq, all gene counts were then imported into the R package “EdgeR” and FPKM normalization size factors were calculated to normalize samples for differences in library size. Data were then imported into the R package “Limma” for the FPKM size factors and count analysis. The differential expression analysis was then performed by the “DESeq2” and the results were filtered for only those genes with Benjamini-Hochberg false-discovery rate adjusted *P* values less than or equal to 0.05. The GSEA were performed using the R package “msigdbr” (version 7.5.1) and “fgsea” (version 1.26.0). The packages “clusterProfiler”, “DOSE”, “org.Mm.eg.db” were applied for GO analysis. Significance was defined as |NES| < 1 or the adjusted q-value (FDR) < 0.25.

**Abbreviations used:** Yy1, Yinyang1; ROS, reactive oxygen stress; RT-qPCR, quantitative real-time polymerase chain reaction; WB, western blotting; H&E, hematoxylin-eosin; TEM, transmission electron microscopy; GSEA, gene set enrichment analysis; GO, gene ontology; NES, normalized enrichment score; OCR, oxygen consumption rate; ChIP, chromatin immunoprecipitation; EMSA, Electrophoretic Mobility Shift Assay; TSS, transcription-start-site; WT, wild type; TA, anterior tibial muscle; SDH, succinate dehydrogenase; ETC, electron transport chain; TGF, transforming growth factor; OXPHOS, oxidative phosphorylation; MOI, multiplicity of infection; AAV, adeno-associated virus; FBS, fetal bovine serum; PVDF, polyvinylidene difluoride; PFA, paraformaldehyde; CSA, cross-section area; ANOVA, analysis of variance.

**Authors’ contributions:**

S.C. and H.-L.Z. were responsible for the conception and designed the research; S.C., J.D.C., T.T.H. conducted the animal experiments and in *vitro* study. S.C., J.D.C., C.W., W.G.H. and Z.J.Y. collected the animal experiment data. S.C. and D.S.W. performed the construction of the plasmid, ChIP and luciferase assay. S.C., T.T.H. and M.T. Y. conducted the statistical analysis and pictures graphing. M.C.L., S.Y.H. and Z.H.H. assists the data collection. S.C. and H.-L.Z. involved in data interpretation and manuscript writing; H.-L.Z. finally approved the manuscript. No conflict of interest exits in the submission of this manuscript, and manuscript is approved by all authors for publication after reading it.

**Data Availability Statement**

Datasets generated during the current study are available from the corresponding author on reasonable request.
