# Supplementary material for: Betaine delays age‐related muscle loss by mitigating Mss51‐induced impairment in mitochondrial respiration via Yin Yang1
Source: J Cachexia Sarcopenia Muscle. 2024 Aug 26;15(5):2104–17. doi: 10.1002/jcsm.13558 (PMC11446699; doi:10.1002/jcsm.13558)
Supplement: Supplementary file 2 — Figure S1. Betaine alleviated skeletal muscle loss (mass, strength, physical performance) during the aging process. (A‐B) Aging‐induced adverse alterations in body composition could be alleviated by betaine treatment. Mean and SD, n = 5–6/group. Betaine partially prevented against (A) decrease in muscle mass and (B) increase in fat mass with age. (C) Betaine alleviated age‐related decline in muscle strength. Mean and SD, n = 6/group. (D) Betaine preserved the ability of muscle performance (running distance, response time to electronic shock) during aging. Mean and SD, n = 4 per group. (E) Representative figures of H&E staining depict that betaine reduced the centralization of muscle nuclei (yellow arrows) and (F) protected against age‐related atrophy in the skeletal muscle cross‐sectional area. n = 4/per group. *: P < 0.05; **: P < 0.01; ***: P < 0.001. Figure S2. Bioinformatic analysis of pathways altered during aging and betaine treatment in skeletal muscle. (A‐B) GO analysis indicated a higher enrichment of genes related to electron carrier activity and fatty acid beta‐oxidation in the YOU or BET groups. n = 4–6/group. (C) The top 50 genes among the three groups were employed for GSEA. Pathways clustered into the muscle system process in the YOU and BET groups. n = 4–6/group. (D) The volcano plot demonstrates that compared with the OLD group, Mss51 mRNA expression was significantly lower in the YOU group. n = 4–6/group. Figure S3. (A) C2C12 cells stably overexpressing Mss51 were established. The efficiency of lentivirus vector transduction into the C2C12 cells was demonstrated by GFP expression. (B) Western blotting was performed to evaluate the efficiency of Mss51 transfection in C2C12 cells. (C) Western blotting was performed to evaluate the efficiency of Yy1 OE (~50%) and KD (~80%) in C2C12 cells. (D‐E) Western blotting was performed to evaluate the efficiency of AAV‐mediated Yy1 OE (~145‐folds) and KD (~82%) in vivo.. [file JCSM-15-2104-s004.docx]

**Supplemental Figures**

**
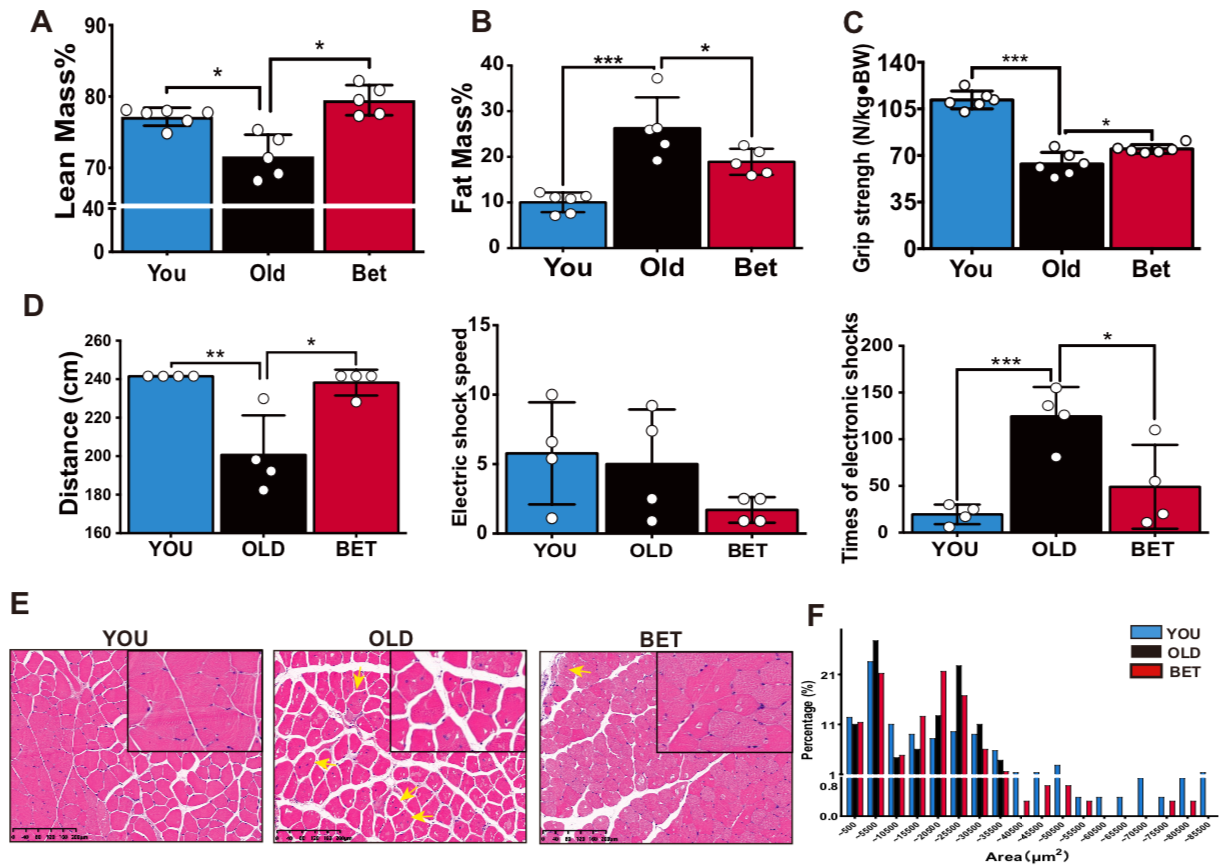
Supplemental Figure1 Betaine alleviated skeletal muscle loss (mass, strength, physical performance) during the aging process.**

(**A-B**) Aging-induced adverse alterations in body composition could be alleviated by betaine treatment. Mean and SD, n=5-6/group. Betaine partially prevented against (**A**) decrease in muscle mass and (**B**) increase in fat mass with age. (**C**) Betaine alleviated age-related decline in muscle strength. Mean and SD, n=6/group. (**D**) Betaine preserved the ability of muscle performance (running distance, response time to electronic shock) during aging. Mean and SD, n=4 per group. (**E**) Representative figures of H&E staining depict that betaine reduced the centralization of muscle nuclei (yellow arrows) and (**F**) protected against age-related atrophy in the skeletal muscle cross-sectional area. n=4/per group. *: *P*<0.05; **: *P*<0.01; ***: *P*<0.001.


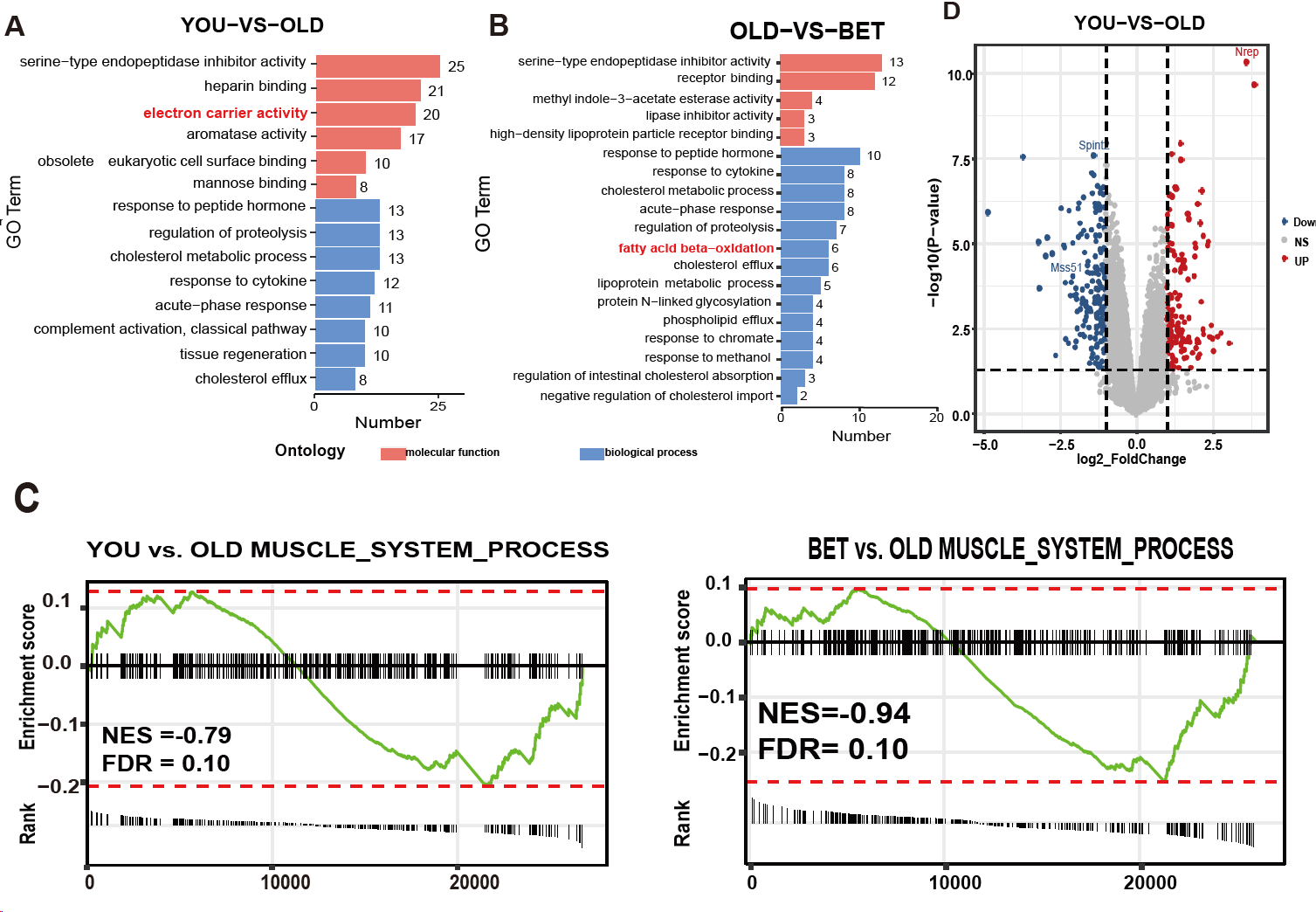
**Supplemental Figure2 Bioinformatic analysis of pathways altered during aging and betaine treatment in skeletal muscle.**

(**A-B**) GO analysis indicated a higher enrichment of genes related to electron carrier activity and fatty acid beta-oxidation in the YOU or BET groups. n=4-6/group. (**C**) The top 50 genes among the three groups were employed for GSEA. Pathways clustered into the muscle system process in the YOU and BET groups. n=4-6/group. (**D**) The volcano plot demonstrates that compared with the OLD group, *Mss51* mRNA expression was significantly lower in the YOU group. n=4-6/group.

**
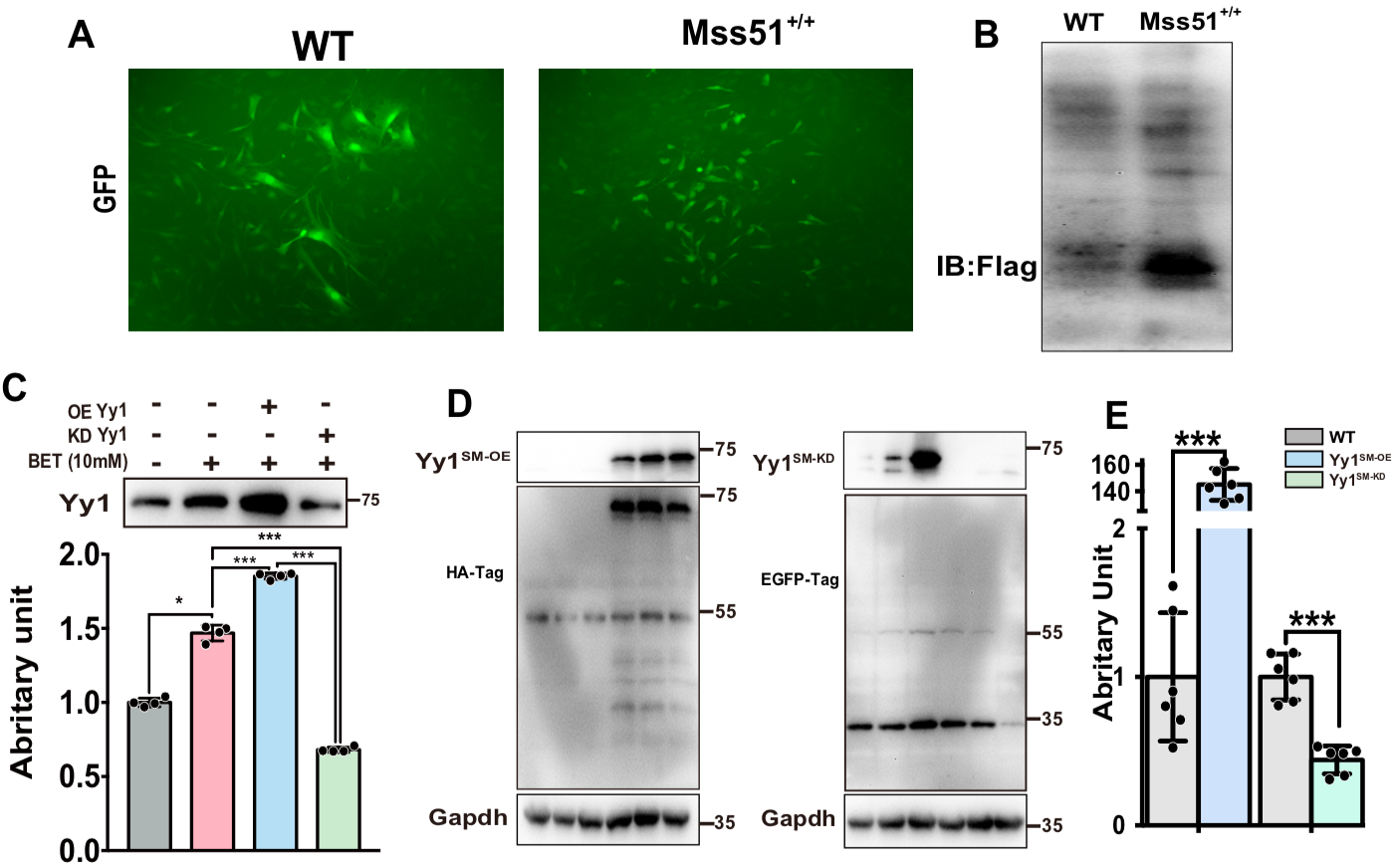
Supplemental Figure3 (A)** C2C12 cells stably overexpressing Mss51 were established. The efficiency of lentivirus vector transduction into the C2C12 cells was demonstrated by GFP expression. (**B**) Western blotting was performed to evaluate the efficiency of Mss51 transfection in C2C12 cells. (**C)** Western blotting was performed to evaluate the efficiency of Yy1 OE (~50%) and KD (~80%) in C2C12 cells. (**D-E)** Western blotting was performed to evaluate the efficiency of AAV-mediated Yy1 OE (~145-folds) and KD (~82%) *in vivo.*
