# Supplementary material for: Betaine delays age‐related muscle loss by mitigating Mss51‐induced impairment in mitochondrial respiration via Yin Yang1
Source: J Cachexia Sarcopenia Muscle. 2024 Aug 26;15(5):2104–17. doi: 10.1002/jcsm.13558 (PMC11446699; doi:10.1002/jcsm.13558)
Supplement: Supplementary file 3 — Table S1. Primer sequences used to amplify DNA. Table S2. Plasmid sequences of lentiviral vectors of Mss51. Table S3. Yy1 plasmid sequences for Dual‐luciferase report assay. Table S4. Mss51 promoter plasmid sequences for Dual‐luciferase report assay. Table S5. Probe design for EMSA. [file JCSM-15-2104-s002.docx]

**Supplemental Tables**

**Supplemental Table 1** Primer sequences used to amplify DNA

| Primer Name | Sequences |
| --- | --- |
| mouse *Atp2a* forward | TGGAACAACCCGGTAAAGAGT |
| mouse *Atp2a* reverse | CACCAGGGGCATAATGAGCAG |
| mouse *Atp5a* forward | CTGCCTTACCAGTCATTGAAAC |
| mouse *Atp5a* reverse | TCCGTCAGTGATGGAAATAACA |
| mouse *Sdha* forward | GGAACACTCCAAAAACAGACCT |
| mouse *Sdha* reverse | CCACCACTGGGTATTGAGTAGAA |
| mouse *Ucp2* forward | ATGGTTGGTTTCAAGGCCACA |
| mouse *Ucp2* reverse | CGGTATCCAGAGGGAAAGTGAT |
| mouse *Ndufa9* forward | GAAGTACATCTTTGGCATGACC |
| mouse *Ndufa9* reverse | GGCATCACGTCTGAGATATGTA |
| mouse *Pgc 1α* forward | TATGGAGTGACATAGAGTGTGCT |
| mouse *Pgc 1α* reverse | CCACTTCAATCCACCCAGAAAG |
| mouse *Mss51* forward | GAAACCCCCACCAGTGATTCC |
| mouse *Mss51* reverse | CCAAGTGCATCAATGCTAGGG |
| mouse *Stk26* forward | AAGTGCCTGGGATGCAGAATA |
| mouse *Stk26* reverse | TGCTGAGTACGGTTATCAATTCC |
| mouse *Col1a1* forward | GCTCCTCTTAGGGGCCACT |
| mouse *Col1a1* reverse | CCACGTCTCACCATTGGGG |
| mouse *Actc1* forward | CTGGATTCTGGCGATGGTGTA |
| mouse *Actc1* reverse | CGGACAATTTCACGTTCAGCA |
| mouse *Yy1* forward | CAGTGGTTGAAGAGCAGATCAT |
| mouse *Yy1* reverse | AGGGAGTTTCTTGCCTGTCAT |
| mouse *Sp1* forward | GCCGCCTTTTCTCAGACTC |
| mouse *Sp1* reverse | TTGGGTGACTCAATTCTGCTG |
| mouse *Smad3* forward | CACGCAGAACGTGAACACC |
| mouse *Smad3* reverse | GGCAGTAGATAACGTGAGGGA |
| Yy1-ChIP-Mss51 forward | ACGCACGGGTTAAGTGATCT |
| Yy1-ChIP-Mss51 reverse | ACCAAAGGAAAGGACCCACTG |
| mouse *18S* forward | GGGAGCCTGAGAAACGGC |
| mouse *18S* reverse | GGGTCGGGAGTGGGTAATTT |

**Supplemental Table 2** Plasmid sequences of lentiviral vectors of Mss51

| Gene Name | Mss51 | Gene ID | 74843 | |
| --- | --- | --- | --- | --- |
| GenBank ID | NM_029104.1 | Size： | 1341 | |
| Species | Mouse | Clone Site: | EcoRI |  |
| Anti | Amp | | | |
| Vector | GL107 pSLenti-EF1-EGFP-P2A-Puro-CMV-MCS-3xFLAG-WPRE | | | |
| Constructor | pSLenti-EF1-EGFP-P2A-Puro-CMV-Mss51-3xFLAG-WPRE | | | |
| Forward | CMV-F CGCAAATGGGCGGTAGGCGTG | | | |
| Reverse | WPRE-R CATAGCGTAAAAGGAGCAACA | | | |

**Supplemental Table 3** Yy1 plasmid sequences for Dual-luciferase report assay

| Plasmid Name | Yy1 | Gene ID | 22632 | |
| --- | --- | --- | --- | --- |
| GenBank ID | NM_009537.4 | Size： | 1245 | |
| Species | Mouse | Clone Site: | EcoRI |  |
| Anti | Amp | | | |
| Vector | H302 pcDNA3.1(+) | | | |
| Constructor | pcDNA3.1(+)-Yy1 | | | |
| Forward | CMV-F CGCAAATGGGCGGTAGGCGTG | | | |
| Reverse | BGH TAGAAGGCACAGTCGAGG | | | |

**Supplemental Table 4** Mss51 promoter plasmid sequences for Dual-luciferase report assay

| Plasmid Name | promoter(Mss51) | Gene ID | 74843 |
| --- | --- | --- | --- |
| GenBank ID | NM_029104.1 | Size： | 2291 |
| Species | Mouse | Clone Site: | XhoI, HindIII |
| Anti | Amp | | |
| Vector | H352 pGL4.10 | | |
| Constructor | pGL4.10-promoter(Mss51) | | |
| Forward | RVprimer3 CTAGCAAAATAGGCTGTCCC | | |
| Reverse | Luc2-N-R CGTCTTCGAGTGGGTAGAATG | | |

**Supplemental Table 5 Probe design for EMSA**

| Probe Name | Sequences (5’-3’) |
| --- | --- |
| Probe1 forward | TCTTCATGGATAGTTTTACCAAAATGGTGAAATATTTAGATAATTTT |
| Probe1 reverse | AAAATTATCTAAATATTTCACCATTTTGGTAAAACTATCCATGAAGA |
| Mutant Probe1 forward | TCTTCATGGATAGTTTTACCAACATGGTGAAATATTTAGATAATTTT |
| Mutant Probe1 reverse | AAAATTATCTAAATATTTCACCATGTTGGTAAAACTATCCATGAAGA |
