# Supplementary material for: Betaine delays age‐related muscle loss by mitigating Mss51‐induced impairment in mitochondrial respiration via Yin Yang1
Source: J Cachexia Sarcopenia Muscle. 2024 Aug 26;15(5):2104–17. doi: 10.1002/jcsm.13558 (PMC11446699; doi:10.1002/jcsm.13558)
Supplement: Supplementary file 4 — Data S2. Supporting Information. [file JCSM-15-2104-s003.pdf]

Figure 1

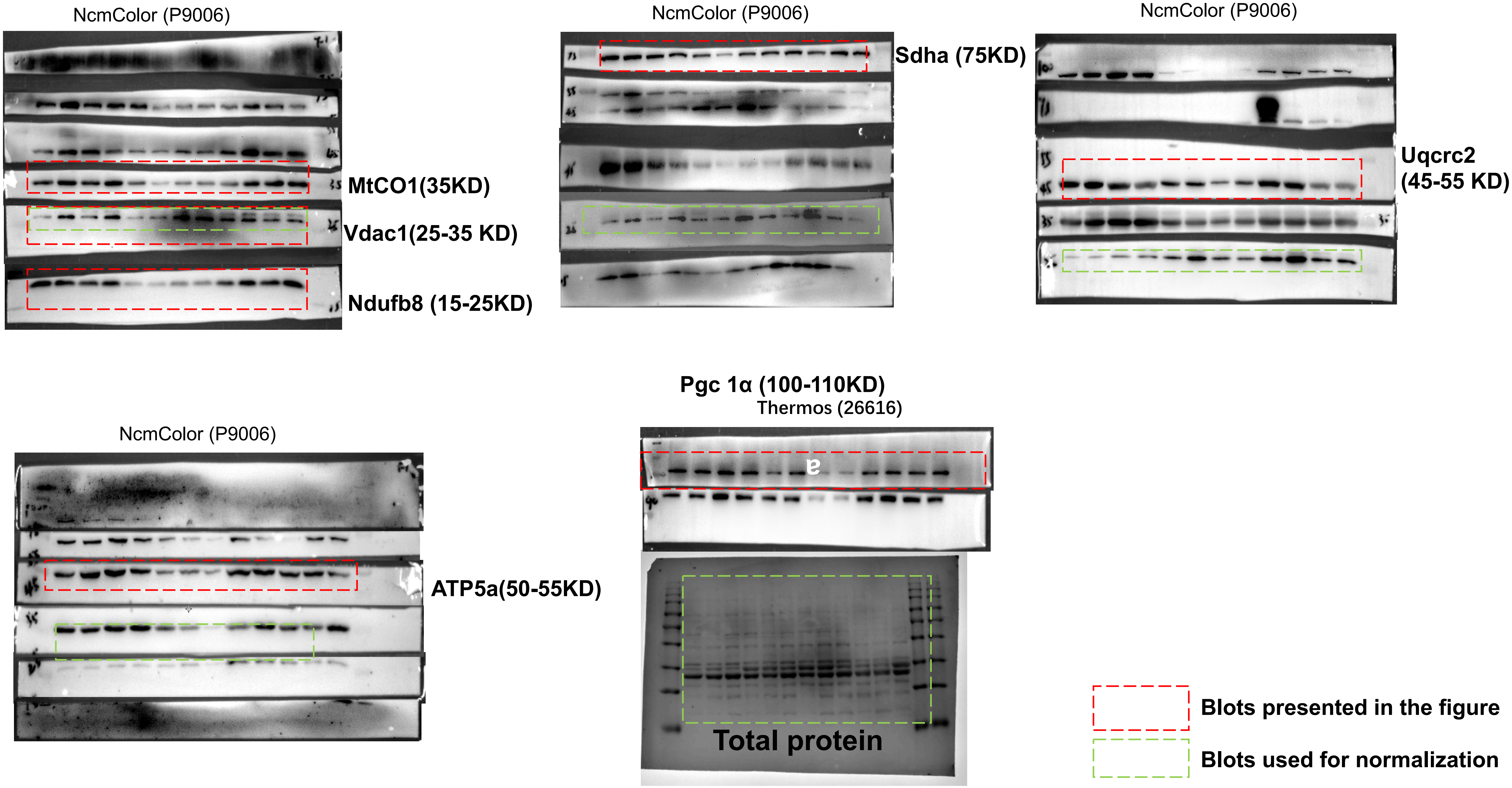
NcmColor (P9006)
MtCO1(35KD)
Vdac1(25-35 KD)
Ndufb8 (15-25KD)
NcmColor (P9006)
Sdha (75KD)
NcmColor (P9006)
Uqcrc2
(45-55 KD)
NcmColor (P9006)
ATP5a(50-55KD)
Pgc 1α (100-110KD)
Thermos (26616)
Total protein
Blots presented in the figure
Blots used for normalization

Figure 3

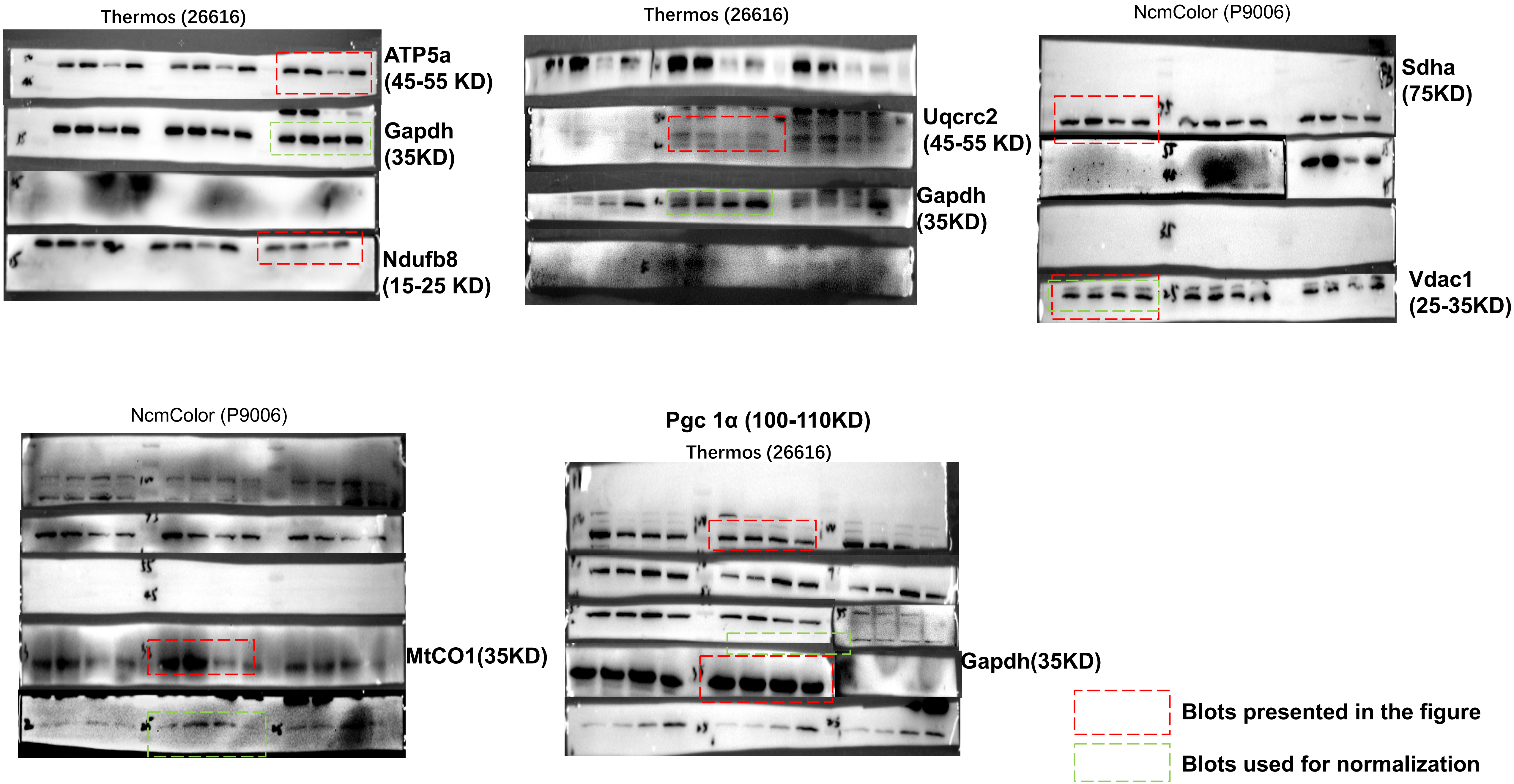
Thermos (26616)
ATP5a (45-55 KD)
Gapdh (35KD)
Ndufb8 (15-25 KD)
Thermos (26616)
Uqcrc2 (45-55 KD)
Gapdh (35KD)
NcmColor (P9006)
Sdha (75KD)
Vdac1 (25-35KD)
NcmColor (P9006)
MtCO1(35KD)
Pgc 1α (100-110KD)
Thermos (26616)
Gapdh(35KD)
Blots presented in the figure
Blots used for normalization

Figure 5

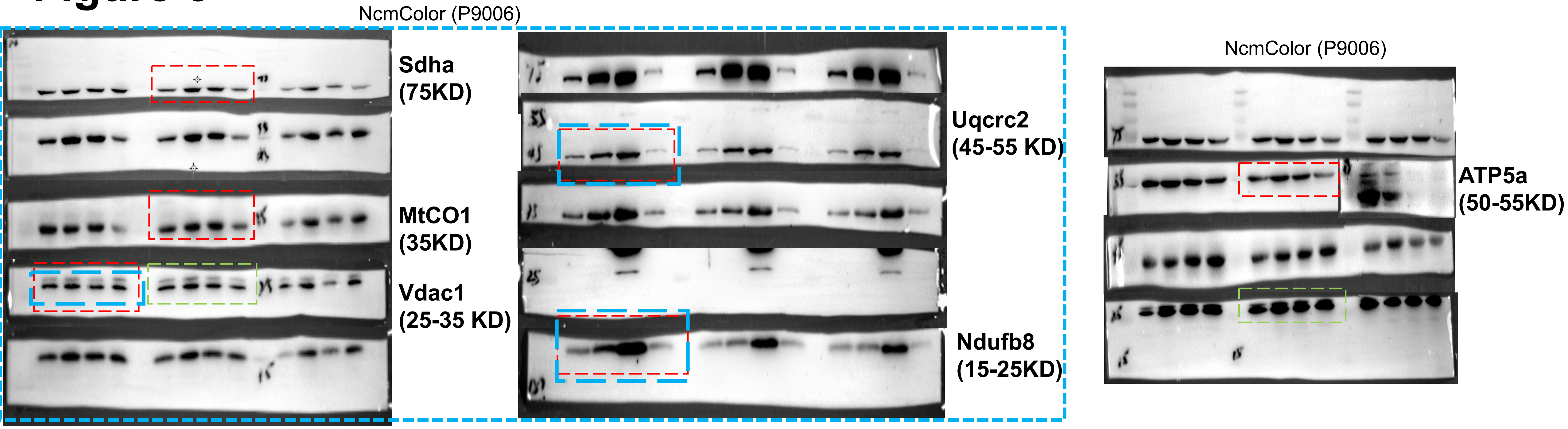

Figure 4

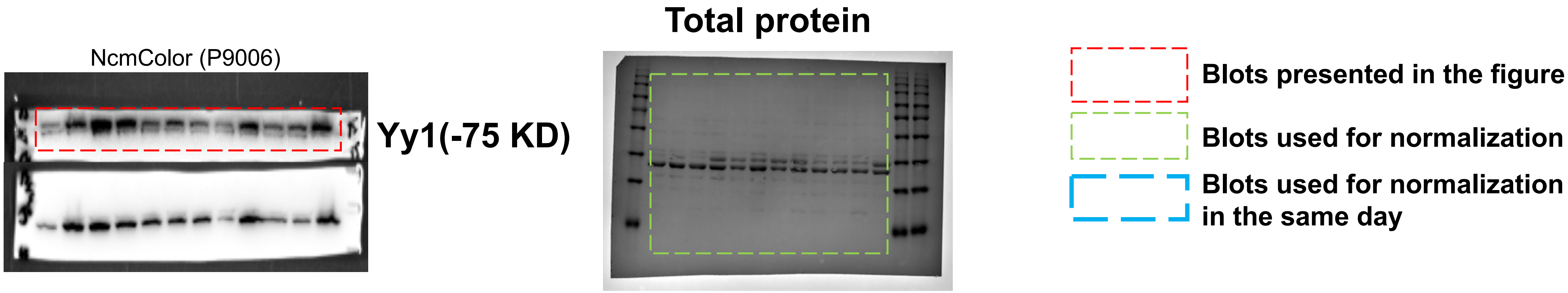

Figure 7

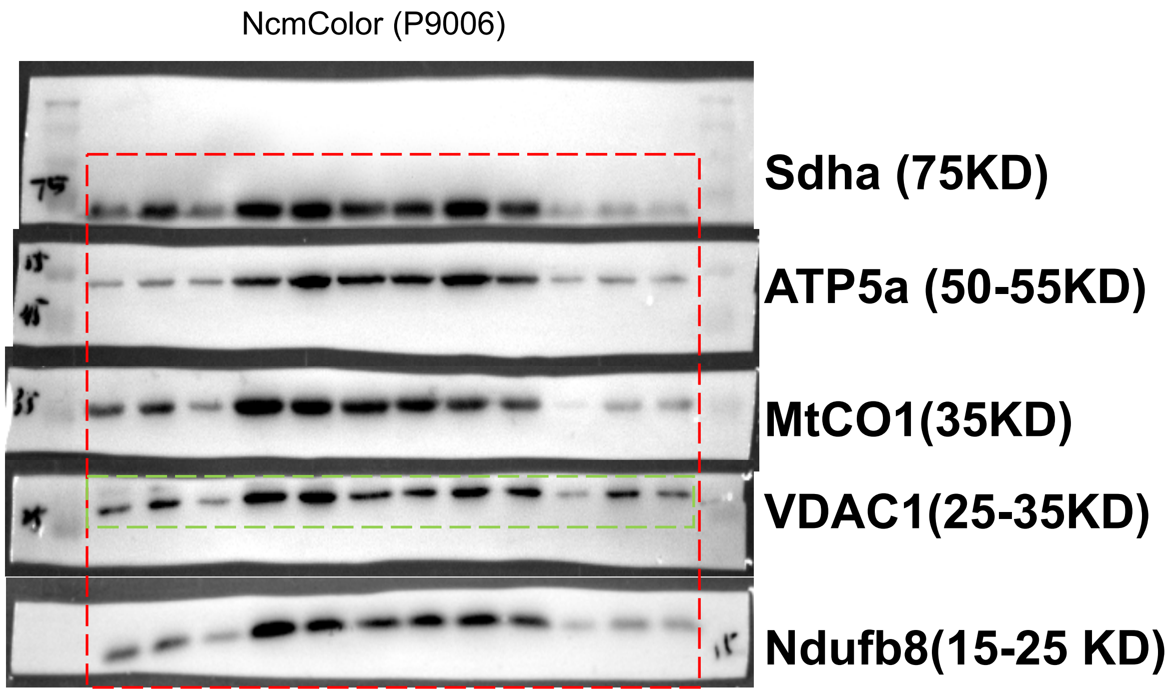
NcmColor (P9006)
Sdha (75KD)
ATP5a (50-55KD)
MtCO1(35KD)
VDAC1(25-35KD)
Ndufb8(15-25 KD)

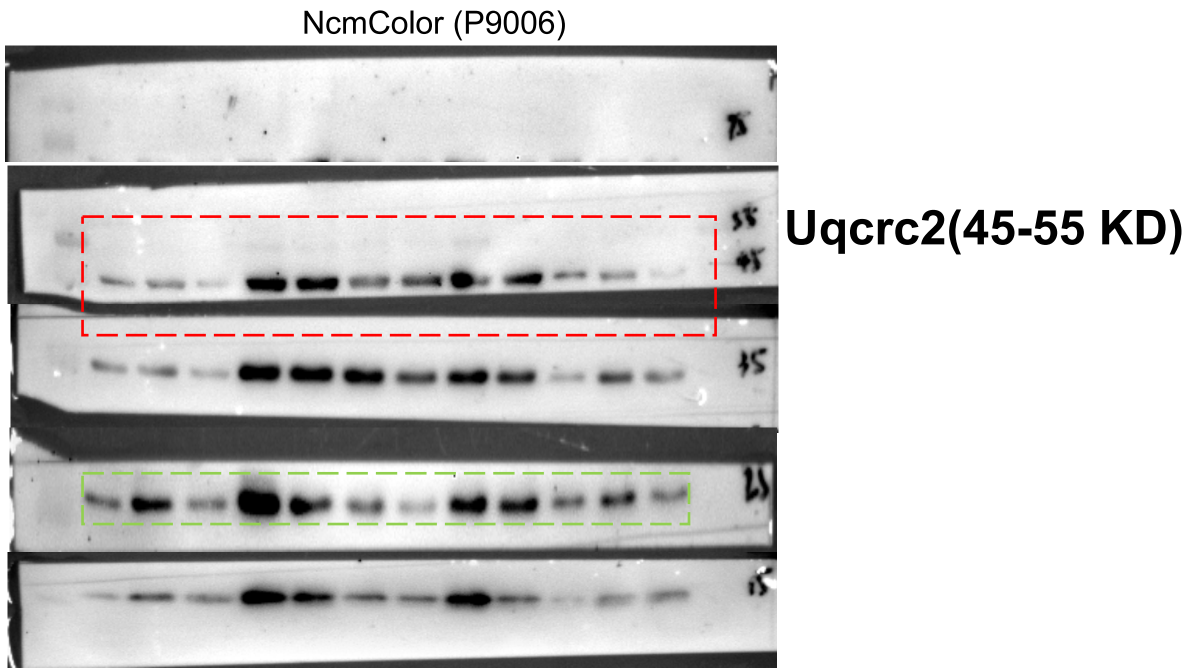
NcmColor (P9006)
Uqcrc2(45-55 KD)

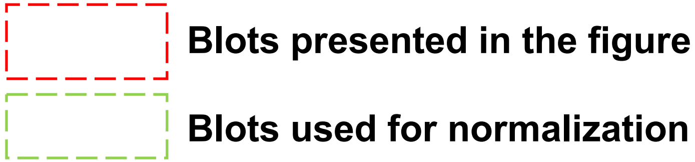
Blots presented in the figure
Blots used for normalization

Fig S3 Yy1 OE/KD

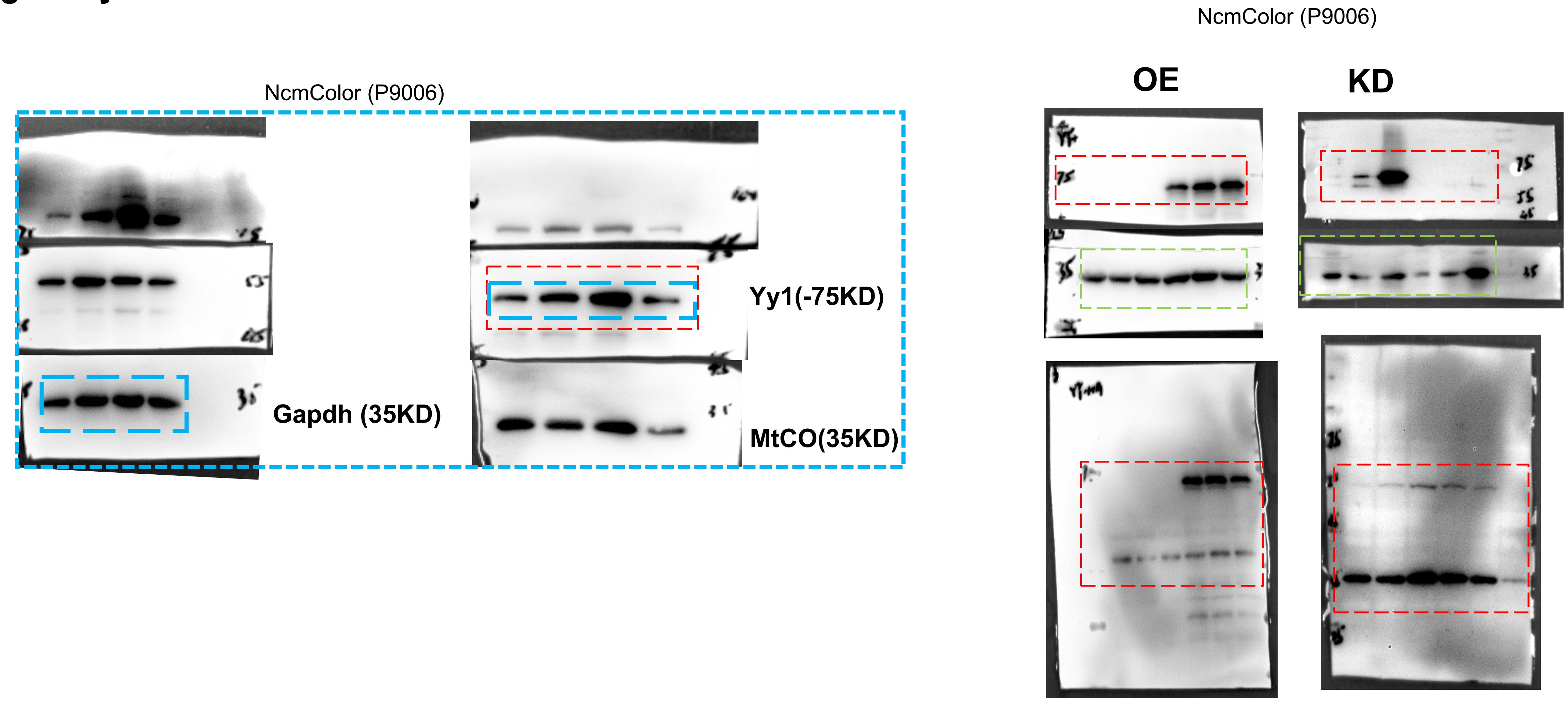

Blots presented in the figure

Blots used for normalization

Blots used for normalization in the same day
